# Novel Nanotherapeutic Systems Based on PEGylated Squalene Micelles for Enhanced In Vitro Activity of Methotrexate and Cytarabine

**DOI:** 10.3390/polym15214225

**Published:** 2023-10-25

**Authors:** Bogdan-Florin Craciun, Isabela-Andreea Sandu, Dragos Peptanariu, Mariana Pinteala

**Affiliations:** Centre of Advanced Research in Bionanoconjugates and Biopolymers, “Petru Poni” Institute of Macromolecular Chemistry, 41A Grigore Ghica Voda Alley, 700487 Iasi, Romania; sandu.isabela@icmpp.ro (I.-A.S.); dragos.peptanariu@icmpp.ro (D.P.)

**Keywords:** PEGylated squalene, drug delivery, methotrexate, cytarabine, antitumoral, controlled release, micelles, MCF-7, HeLa

## Abstract

Nanomedicine has garnered significant attention due to the advantages it offers in the treatment of cancer-related disorders, some of the deadliest diseases affecting human lives. Conventional medication formulations often encounter issues of instability or insolubility in biological environments, resulting in low bioavailability. Nanocarriers play a crucial role in transporting and safeguarding drugs at specific sites of action, enabling gradual release under particular conditions. This study focuses on methotrexate (MTx) and cytarabine (Cyt), essential antitumoral drugs, loaded into PEGylated squalene micellar structures to enhance therapeutic effectiveness and minimize drawbacks. The micelles were prepared using ultrasound-assisted methods in both water and phosphate buffer saline solutions. Evaluation of drug-loaded micelles encompassed parameters such as particle size, colloidal stability, surface charge, morphology, encapsulation efficiency, drug loading capacity, and in vitro release profiles under simulated physiological and tumoral conditions. In vitro cell inhibition studies conducted on MCF-7 and HeLa cell lines demonstrated higher antitumoral activity for the drug-encapsulated micelles compared to free drugs. The encapsulation effectively addressed the burst effect, providing sustained release for at least 48 h while enhancing the drug’s protection under physiological conditions.

## 1. Introduction

Cancer is one of the most life-threatening diseases. The onset of this illness, combined with the suppression of the immune system due to daily stress and an unhealthy lifestyle, not only diminishes the quality of life but, in the absence of effective treatment, can lead to mortality [1]. In recent years, different strategies have been involved in treating or eradicating cancer. Among them, nanomedicine received the greatest attention due to the advantages in nanoformulations of existing therapeutics, which are able to confer superior efficiency and biological peculiarities [2,3,4]. Even newly discovered drugs, such as peptides, siRNA, therapeutic nucleic acids, or proteins, are very unstable or insoluble in the biological environments and present poor bioavailability in common formulations. On this basis, they often require a delivery nanosystem and, therefore, accessing the field of nanotechnology for the improvement of existing issues is recommended [5,6]. It is worth mentioning that in the field of anticancer drug delivery, due to the high toxicity of administrated drugs, nanocarriers should transport and protect innovative or classic drugs during transportation to a specific site of action. At this site, the loaded active molecules should be released under specific conditions, without causing toxicity due to the breakdown of the nanocarrier [7,8].

The excellent properties of nanotherapeutics (e.g., low toxicity, improved bioavailability, and enhanced therapeutic effect) make them ideal candidates for clinical trials or clinical use [9,10]. These are valuable properties due to their reduced size (10–100 nm), high specific surface area, and permissive functionalization of the surface compared with conventional drugs [11]. The drug nanotherapeutic carriers, especially polymer–drug conjugates, dendrimers, liposomes, polymeric micelles, and nanoparticles, are the main classes of compounds investigated for drug delivery and are extensively studied for cancer therapies [12]. Nanotherapeutic development, however, encountered some major challenges, such as “Burst release”, which is one of the most severe limitations of both conventional drugs and nanotherapeutics and consists of the instant release of the majority of the loaded drug [13]. Moreover, the encapsulation of drugs with poor solubility leads to colloidal instability; because of this, organic co-solvents must be utilized during the formulation process. However, this results in a greater level of toxicity, which can only be reduced by performing additional synthetic stages [14]. Although there are many nanoformulations reported in the literature, only a few numbers are chosen for clinical studies. This is mostly due to a number of issues that arise after the translation of in vitro to in vivo experiments, which are related to biological barriers that are very difficult to overcome [15,16].

Among the large area of nanomaterials, the lipid-based ones are intensely studied and represent the majority of drug vehicles that were approved for clinical use due to their capacity to encapsulate a higher amount of drugs, their ability to target a specific tissue or cell line, and their degradability under various stimuli without toxic degradation compounds [16,17]. Usually, triglycerides or phospholipids, fatty acids, and cholesterol derivatives are used, due to their improved biocompatibility. The proof of the above-mentioned statement is that the currently available nanotherapeutics used in clinical trials are lipid-based liposomal doxorubicin (Myocet^®^) [18], PEGylated liposomal doxorubicin (Caelyx^®^) [19], and the liposomal formulation of amphotericin B (Ambisome^®^) [20], as well as a docosahexaenoic acid (omega-3 fatty acid) conjugate of paclitaxel (Taxoprexin) [21], an elaidic acid conjugate of cytarabine (Elacyt) [22], and a cardiolipin conjugate of gemcitabine (Neopharm) [23], which reached Phase I/II clinical trials. One of the lipids that proved its value in the formation of lipid–drug nanoassemblies used in different combinations of therapeutic conjugates is squalene [24,25,26]. Being a natural lipid widespread in nature (e.g., shark liver oil), squalene is a precursor for cholesterol and other triterpenes’ biosynthesis. In the human body, squalene is distributed in different organs, and the highest concentration is in the epidermal tissue [27]. Squalene is biocompatible and well tolerated by organisms either ingested or administrated intravenously [28,29]. Furthermore, this natural triterpene is highly hydrophobic. When combined with a hydrophilic component, such as polyethylene glycol (commonly chosen due to its biocompatibility, biodegradability, and hydrophilic properties), it can be used to create an amphiphilic copolymer. This copolymer has the ability to self-assemble in water into micellar structures, forming unique and stable core–shell structures above the critical micelle concentration (CMC) [24,30,31,32,33]. The hydrophobic core promotes the solubilization of water-insoluble drugs, protecting them from degradation by harsh environments, whereas their outer hydrophilic shell can reduce the binding of plasma proteins and minimize nonspecific uptake by the reticuloendothelial system, prolonging their blood circulation time [34].

Methotrexate (MTx) and cytarabine (Cyt) played an instrumental role in the treatment of various types of cancer and leukemia [35,36,37,38,39,40,41,42]. However, the applications of both drugs are limited due to their inadequate solubility, toxic side effects, nonspecific delivery, and low permeability [43,44]. To improve their therapeutic efficacy and reduce their multiple drawbacks, the current study focused on obtaining MTx- and Cyt-loaded nanotherapeutics based on PEGylated squalene micellar architectures to characterize and evaluate them for antitumour applications. First, the PEGylated squalene micellar nanoassemblies were obtained via the ultrasound-assisted method in ultra-pure water or a phosphate buffer saline solution (PBS). Then, MTx-loaded micelles were obtained through the ultrasound-assisted dropping method; meanwhile, Cyt-loaded micelles were obtained with the ultrasound-assisted solvent evaporation method. The obtained drug-loaded micelles were characterized and evaluated to determine particle sizes, colloidal stability, surface charge, morphology, encapsulation efficiency, drug loading capacity, and in vitro release profiles in simulated physiological and tumoral conditions, as well as the release kinetics on different mathematical models. The in vitro cell inhibition studies of the obtained nanotherapeutics were evaluated on the MCF-7 and HeLa cell lines via a CellTiter-Glo assay. Apoptosis was confirmed by luminescence measurements with a filter-based multi-mode microplate reader. The encapsulation of the commercially available drugs in PEGylated squalene micelles proved to be a suitable approach to overcome the burst effect within 4 to 8 h with a sustained release pattern during at least 48 h together with an improved protection for the transported drug in physiological conditions.

## 2. Materials and Methods

### 2.1. Materials

Squalene (SQ) ≥ 98%, tetrahydrofuran (THF), N-bromosuccinimide (NBS) ReagentPlus 99%, sodium bicarbonate (NaHCO_3_) ≥ 99.5%, di-tert-butyl decarbonate (Boc_2_O) 97%, and N,N-dimethylformamide (DMF) anhydrous, 99.8% were purchased from Sigma-Aldrich, Germany. Diethyl ether (Et_2_O) ≥ 99.8%, methanol (MeOH) CHROMASOLV ≥ 99.9%, and ethyl acetate (EtOAc) ≥ 99.5% were acquired from Honeywell|Riedel-de Haen, Saint-Germain-en-Laye, France. Petroleum ether 40–60 °C ACS > 99.9%, sodium chloride (NaCl) ACS > 99%, and 1,4-dioxane AnalaR NORMAPUR were purchased from VWR Chemicals, Leuven, Belgium. Sodium phosphate dibasic dihydrate (Na_2_HPO_4_**·**2H_2_O) puriss 98.5–101% and sodium phosphate monobasic monohydrate (NaH_2_PO_4_**·**H_2_O) ACS ≥ 98% were purchased from Honeywell|Fluka, Mosbach, Germany. Potassium chloride (KCl) ReagentPlus ≥ 98% (Honeywell|Fluka, Acton, MA, USA), anhydrous sodium sulphate (Na_2_SO_4_) ACS ≥ 99% (Honeywell|Fluka, Pune, India), hydrochloric acid (HCl) puriss, fuming ≥ 37% (Honeywell|Fluka, Vienna, Austria), methylene chloride (DCM) puriss ≥ 99.9% (Honeywell, Riedel-de Haen, Germany), potassium carbonate (K_2_CO_3_) ACS ≥ 99% (Sigma-Aldrich, Madrid, Spain), anhydrous magnesium sulfate (MgSO_4_) ≥ 99.5% (Sigma-Aldrich, Tokyo, Japan), α,ω-bis(3-aminopropyl) poly(ethylene glycol) (Mn~1500 Da) (NH_2_-PEG_1500_-NH_2_) (Sigma-Aldrich, Košice, Slovakia), N-hydroxysuccinimide (***NHS***) 98% (Sigma-Aldrich, Shanghai, China), potassium dichromate (K_2_Cr_2_O_7_) ReagentPlus ≥ 99.5% (Sigma-Aldrich, Moskow, Russia), 1-(3-dimethylaminopropyl)-3-ethylcarbodiimide hydrochloride (EDC-HCl) purum ≥ 98% (Fluka, London, UK), periodic acid (HIO_4_) 99% (Alfa Aesar, Karlsruhe, Germany), n-hexane 99% (J.T.Baker, Hamburg, Germany), triethylamine (TEA) > 99% (TCI, Hamburg, Germany), pyrene 98% (Thermo Scientific, Shanghai, China), sulfuric acid (H_2_SO_4_) 96% (Chemical Company, Iasi, Romania), methotrexate (MTx) Pharmaceutical Secondary Standard C.R.M. (Supelco, Shanghai, China), cytarabine (Cyt) ≥ 98% (Merck EMD Millipore, Burlington, MA, USA), and double-distilled water (dd-H_2_O) were obtained with an FiSTREEM Cyclon WSC044.MH3.4 system, and ultra-pure water (mQ-H_2_O) was obtained with a TKA-GenPure 08.2204 system. The cervical cancer cell line (HeLa) and human breast cancer cell line (MCF-7) were purchased from CLS Cell Lines Service. A Penicillin–Streptomycin–Amphotericin B mixture was purchased from Lonza. Fetal bovine serum was from Sigma Aldrich, Dulbecco′s Modified Eagle Medium with low glucose, no nucleosides (DMEM), and TrypLE Express were from Gibco. Phosphate buffered saline (PBS) was from Invitrogen. The CellTiter-Glo Luminescent Cell Viability Assay was from Promega, and 96-well white opaque tissue culture-treated plates were from Perkin Elmer.

All the chemicals were used, as they were purchased without any purification.

### 2.2. Methods

#### 2.2.1. Nuclear Magnetic Resonance (NMR)

^1^H-NMR and ^13^C-NMR spectra were recorded on a Bruker Avance III 400 instrument operated at 400 and 100 MHz, respectively, at room temperature (23 °C). Chemical shifts are reported in ppm using tetramethylsilane (TMS) as an internal standard. Samples were prepared by solubilizing approximately 40 mg of the completely dried compound in 0.6 mL of deuterated solvent (CDCl_3_). The obtained spectra were edited with MestReNova 6.0.2–5475 from Mestrelab Research S.L [45].

#### 2.2.2. Attenuated Total Reflection Fourier-Transform InfraRed Spectroscopy (ATR-FTIR)

ATR-FTIR spectra were recorded on an IRTracer-100 Fourier-Transform Infrared Spectrophotometer from Shimadzu equipped with a GladiATR Single Reflection ATR module from PIKE Technologies. The obtained IR spectra were processed with the LabSolutions IR software v2.3 from Shimadzu and further edited with Origin Pro v2020b software from OriginLab [46].

#### 2.2.3. Matrix-Assisted Laser Desorption/Ionization Time-of-Flight Mass Spectrometry (MALDI-TOF MS)

The MALDI-TOF MS analysis was achieved using the rapifleX^®^ MALDI-TOF/TOF system from Bruker Daltonics equipped with a Smartbeam 3D laser. FlexControl (Bruker Daltonics, Version 4.0) was used to optimize and acquire data. The samples were dissolved in chloroform and diluted in a 50% chloroform/50% acetonitrile mixture. For the MALDI matrix solution, 20 mg of 2,5-dihydroxybenzoic acid (DHB, Sigma, Buchs, Switzerland) was dissolved in 1 mL acetonitrile. Then, the MALDI matrix solution and sample solution were mixed in 2:1, 5:1, and 10:1 volume ratios and finally, 1 µL from each final solution was deposited on an MTP 384 ground steel plate. The samples were allowed to dry in ambient conditions (23 °C), and the MS spectra were obtained using the following parameters: positive ion polarity in reflector mode, mass scan range (*m*/*z* 300–3200), digitizer (2.5 GHz), detector voltage (2028 V), 1000 shots per pixel, and 10 kHz laser frequency. The laser power was set at 40 to 80% of the maximum, and 8000 laser shots were accumulated for each spectrum. Mass calibration of MALDI-TOF/TOF MS was performed by the peptide mixture standard solution (Bruker Daltonics, Bremen, Germany).

#### 2.2.4. Electrospray Ionization Mass Spectrometry (ESI-MS)

Data acquisition and analysis were performed using an Agilent 6500 Series Accurate-Mass Quadrupole Time-of-Flight (Q-TOF) LC/MS instrument. The SQ-COOH solution was introduced into the electrospray ion source (ESI) via a syringe pump at a flow rate of 0.03 mL/min. The ESI-Q/TOF MS conditions were set as follows: electrospray ionization (negative ion mode); drying gas (N_2_) flow rate 8.0 L/min; drying gas temperature 325 °C; nebulizer pressure 40 psi; capillary voltage 4200 V; and fragmentation voltage 175 V. The full-scan mass spectra of the investigated compounds were acquired in the range of *m*/*z* 100–1500. Data were collected and processed using MassHunter Workstation Software Data Acquisition for 6200/6500 Series, version B.07.00 (Agilent Technologies, Inc., Santa Clara, CA, USA).

#### 2.2.5. Critical Micelle Concentration (CMC)

The critical micelle concentration (CMC) of the SQ-PEG_1500_-NH-Boc copolymer in PBS aqueous solutions with pH values of 6.5 and 7.4 was assessed using pyrene as a fluorescent probe on a FluoroMax-4 fluorescence spectrophotometer (HORIBA Scientific) [31]. To determine the CMC, various concentrations of SQ-PEG_1500_-NH-Boc solutions were prepared, ranging from 3.24 × 10^−8^ M to 13 × 10^−3^ M, by combining the PBS aqueous solution with pH values of 6.5 and 7.4 (1 mL) with pyrene (1.0 × 10^−7^ M in dd-H_2_O) (1 mL). The resulting mixtures were magnetically stirred at 60 °C for 90 min and then allowed to equilibrate to room temperature (23 °C) overnight under continuous stirring. The fluorescence spectra of pyrene were measured by exciting it at 334 nm, and the emission spectra were recorded in the range of 349–600 nm (averaging 3 scans) with an integration time of 0.1 s and slit widths of 2 nm (entrance) and 2 nm (exit).

To process the obtained spectra, baseline subtraction and normalization were performed using Origin Pro v2020b software from OriginLab [46]. Subsequently, the ratio of fluorescence intensities at 380 nm and 410 nm (I_3_/I_E_) were calculated for each sample, and the data were plotted against the logarithmic concentration of the corresponding samples. Finally, the CMC value of the SQ-PEG_1500_-NH-Boc copolymer was determined by fitting the plotted data using Boltzmann sigmoidal fitting.

#### 2.2.6. Ultraviolet–Visible Spectroscopy (UV-Vis)

Lambda 35 UV-Vis spectrophotometer from Perkin Elmer was used to acquire the absorbance spectra. Aqueous solutions containing the studied compounds at the desired concentrations were evaluated in quartz cuvettes with a 10 mm optical path. The UV-Vis spectra were recorded in double beam mode, with the solvent used to dilute the studied samples serving as a blank. Origin Pro v2020b software from OriginLab was used to process the acquired absorption spectra [46].

#### 2.2.7. Dynamic Light Scattering (DLS)

The PEGylated squalene blank micelles (*SQ-PEG_1500_-NH-Boc*), MTx encapsulated micelles (*SQ-PEG_1500_-NH-Boc*/*MTx*), and Cyt encapsulated micelles (*SQ-PEG_1500_-NH-Boc*/*Cyt*) mean size and zeta potential were determined using a Beckman Coulter DelsaNano C Submicron Particle Size Analyzer equipped with twin 30 mW laser diodes emitting at 658 nm. The Z-potential was measured using electrophoretic light scattering (ELS), which detects the electrophoretic mobility of charged particles under an applied electric field. The measurements were made at 25 °C, and the result was presented as an average of three readings. The analysis mode used the Smoluchowski equation. Each sample was dispersed in PBS at pH values of 6.5 or 7.4 to obtain the concentrations in a final volume of 2 mL. Size measurements were achieved using a 10 mm Size Glass Cell module (PS cuvette) with the following software settings: accumulation times—70, scattering angle—165°, correlation method—TD, attenuator 1–100%, pinhole—100 μm, and noise threshold—0.3%, and 3 measurements were performed for each sample. Zeta measurements were recorded using a Flow Cell module with the following software settings: accumulation times—50 (10 accumulations in 5 different points), scattering angle—15°, correlation method—TD, attenuator 1–5.23%, attenuator 2–0.4%, and pinhole—100 μm. The obtained results were processed with Delsa Nano Software Version 3.73 from Beckman Coulter Inc. (Brea, CA, USA).

#### 2.2.8. Scanning Transmission Electron Microscopy (STEM)

STEM analysis was performed using a Verios G4 UC Scanning Electron Microscope (Thermo Scientific, Brno, Czech Republic) functioning in STEM Mode at 30 kV, with a STEM 3+ detector. The investigated samples were prepared by depositing the diluted sample on 400 Mesh carbon-coated copper grids (TED PELLA) and air-dried for 24 h in dust-free conditions at ambient temperature (23 °C). The obtained images were processed with ImageJ 1.48r software [47].

#### 2.2.9. Quantification of Encapsulation Efficiency and Loading Capacity

The encapsulation efficiencies (%EE) and drug loading capacities (%DL) of the drugs (MTx and Cyt) loaded into SQ-PEG_1500_-NH-Boc micelles were determined using a UV-vis spectrophotometer at 303 nm (MTx) and 272 nm (Cyt) based on standard calibration curves obtained from MTx and Cyt in PBS with pH values of 7.4 and 6.5. The %EE and %DL were calculated according to Equations (1) and (2) as follows:

(1) $$\%EE=\frac{Weight\;of\;encapsulated\;drug\;\left(mg\right)}{Weight\;of\;initial\;drug\;\left(mg\right)}\times 100$$

(2) $$\%DL=\frac{Weight\;of\;encapsulated\;drug\;(mg)}{Weight\;of\;micelles\;(mg)}\times 100$$

#### 2.2.10. Phosphate Buffer Saline (PBS) Solution Preparation

The PBS solutions with pH values of 6.5 and 7.4 were prepared according to modified protocols published elsewhere [48,49].

#### 2.2.11. In Vitro Drug Release Studies

To evaluate the in vitro release behaviour of MTx and Cyt drugs from MTx- and Cyt-loaded SQ-PEG_1500_-NH-Boc micelles, the common dialysis method [50,51] in PBS media with different pH values (tumoral pH—6.5 and physiological pH—7.4) at 39 °C and 37 °C, respectively, was used. Briefly, 2 mL of MTx- and Cyt-loaded SQ-PEG_1500_-NH-Boc micelles were placed in dialysis bags (MWCO 10 kDa), sealed at both ends with Teflon threads, and immersed completely into 40 mL of release media. The release systems were placed on a heated multi-post magnetic stirrer and stirred at 100 rpm at 37 °C and 39 °C, respectively. At predetermined time points (0, 1, 2, 4, 8, 24, 48, 72, 144, 216, and 360 h), 2 mL of the release media was withdrawn for analysis, and an equal volume of fresh media was replenished to maintain a constant volume. The drug release studies were performed for 216 h (MTx drug) and 360 h (Cyt drug), and all release samples were performed in triplicate. The amount of MTx and Cyt released from the studied systems was quantified by UV-vis absorption at wavelengths of 303 nm for MTx and 272 nm for Cyt. The cumulative release of the MTx and Cyt was calculated using an equation published elsewhere [52]. For comparison, similar release experiments were performed with the same amount of free MTx and Cyt as found in MTx- and Cyt-loaded micelles.

#### 2.2.12. Drug Release Kinetics

The release kinetics of MTx- and Cyt-loaded SQ-PEG_1500_-NH-Boc micelles were determined by fitting the in vitro release experimental data to the following mathematical models: zero-order, first-order, Higuchi, Hixson–Crowell, and Korsmeyer–Peppas, as described elsewhere and presented in the Supplementary Materials [52,53].

#### 2.2.13. Cell Cultures for the In Vitro Experiments

HeLa and MCF-7 cells were cultivated using complete DMEM (low glucose, without nucleosides) containing 5% fetal bovine serum and a 1% Penicillin–Streptomycin–Amphotericin B mixture at 37 °C under a humidified atmosphere with 5% CO_2_. The medium was changed every 3–4 days, and the cells were maintained in culture dishes until they reached subconfluency. The TrypLE express dissociation reagent was used for passing the cells. Stock solutions for treatment were prepared with PBS, and working solutions were obtained by diluting the stock solutions with complete DMEM. Control cells were treated only with a complete cell culture medium.

#### 2.2.14. In Vitro Cytotoxicity Studies

A CellTiter-Glo Luminescent Cell Viability Assay was used to investigate the in vitro inhibition activity of nanotherapeutics. Briefly, MCF-7 cells at 7 × 10^3^ cells/well and HeLa cells at 10 × 10^3^ cells/well in 100 μL DMEM/well and incubated for 24 h. After 24 h, the culture medium was replaced with working solutions, and the plates were incubated for 48 and 72 h. Before reading the results, the plates were removed from the incubator and kept at room temperature for 30 min for equilibration. Then, a 100 µL/well of CellTiter-Glo reagent was added. The plates were stirred for 2 min and then incubated for 15 min at room temperature. In the final step, the luminescence was measured with a FLUOstar Omega Filter-based multi-mode microplate reader (BMG LABTECH). The cell inhibition is expressed as percentages of the viability of control cells, using Equation (3):

(3) $$Cell\;inhibition\;\left(\%\right)=100-\left(\frac{RLus-RLub}{RLuc-RLub}\right)\times 100$$

where *RLus*, *RLub*, and *RLuc* are relative light units recorded for the sample, blank, and control wells, respectively.

#### 2.2.15. Statistical Analysis

Statistical analyses were performed using GraphPad Prism 6.04 for Windows (GraphPad Software, La Jolla, CA, USA). Student’s *t*-test was applied for comparison between studied nanotherapeutics, with 3 replicates for each group included. Results are presented as means ± standard deviation (SD), and a difference was considered statistically significant when values of calculated probability (*p*) were lower than 0.05.

### 2.3. Experimental Procedures

#### 2.3.1. Synthesis of Squalenic Acid (SQ-COOH)

SQ-COOH was obtained from squalene aldehyde (SQ-CHO) using the previously reported protocols [31,54,55,56]. Briefly, SQ-CHO (2.162 g, 5.62 mmol) was solubilized in Et_2_O under magnetic stirring, and the solution was cooled to 0 °C using an ice bath. Next, K_2_Cr_2_O_7_ (1.65 g, 5.62 mmol) was solubilized in small portions in a solution of H_2_SO_4_ (3.03 mL) in dd-H_2_O (28.9 mL) cooled to 0 °C and added dropwise over the SQ-CHO solution keeping the temperature below 0 °C. The obtained reaction mixture was magnetically stirred for 2h at 0 °C, and the reaction progress was monitored by thin layer chromatography (TLC) using an eluent as the mixture of a petroleum ether, Et_2_O, and MeOH in a volume ratio of 70:23:7. The reaction mixture was diluted with brine until a neutral pH was obtained, and the solution was extracted with Et_2_O (3 × 100 mL). The combined organic layers were washed once with brine (1 × 100 mL), twice with dd-H_2_O (2 × 100 mL), dried over anhydrous Na_2_SO_4_, and concentrated to dryness under reduced pressure using a rotary evaporator (10 mbar, 40 °C). The remaining residue, a greenish-yellow oil, was purified by flash chromatography using a 400 mesh SilicaGel packed column and a petroleum ether:Et_2_O volume ratio of 95:5 as an eluent. SQ-COOH was obtained as a colourless oil yielding 1.5 g (67%).

**^1^H-NMR** (CDCl_3_, 400 MHz), δ (ppm): 5.22–5.06 (m, 5H, -C***H***=), 2.48–2.41 (m, 2*H*, -C***H***_2_-), 2.30 (t, *J* = 7.7 Hz, 2H, -C***H***_2_-), 2.13–1.94 (m, 16H, -C***H***_2_-), 1.68 (s, 3H, -C***H***_3_), 1.61 (d, *J* = 6.9 Hz, and 15H, -C***H***_3_) (Figure S1). **^13^C-NMR** (CDCl_3_, 100 MHz), δ (ppm): 179.76 (-***C***OOH), 135.15 (-***C***(CH_3_)=), 134.91 (-***C***(CH_3_)=), 134.86 (-***C***(CH_3_)=), 132.89 (-***C***(CH_3_)=), 131.25 (H_3_C-***C***(CH_3_)=), 125.37 (-***C***H=), 124.48 (-***C***H=), 124.42 (-***C***H=), 124.28 (-***C***H=), 39.76 (-***C***H_2_-), 39.74 (-***C***H_2_-), 39.55 (-***C***H_2_-), 34.29 (-***C***H_2_-), 32.98 (-***C***H_2_-), 28.27 (-***C***H_2_-), 26.78 (-***C***H_2_-), 26.67 (-***C***H_2_-), 26.63 (-***C***H_2_-), 25.70 (-***C***H_2_-), 17.69 (-***C***H_3_), 16.05 (-***C***H_3_), 16.03 (-***C***H_3_), 16.01 (-***C***H_3_), and 15.93 (-***C***H_3_) (Figure S2). **ATR-FTIR** ν (cm^−1^): 2916 (O-H stretch -COOH), 1707 (C=O stretch -COOH), 1442 (C-C stretch), 1381 (C-H bend alkans), 1298 (C-O stretch -COOH), 1097 (C-H bend alkene), and 935 (O-H bend -COOH), (Figure S3). **ESI-MS:** *m*/*z* calculated: 400.33 [100%] and obtained: 399.28 [SQ-COOH-H]^−^ (single charge deprotonated ion) and 799.59 [2SQ-COOH-H]^−^ (deprotonated dimer of SQ-COOH) (Figure S4).

#### 2.3.2. Synthesis of Amine-PEG_1500_-NH-Boc-Protected (NH_2_-PEG_1500_-NH-Boc)

The protection of a single amino group of H_2_N-PEG_1500_-NH_2_ was accomplished by adapting a reported protocol [57]. Briefly, bis-amine-terminated PEG_1500_ (2 g, 1.33 mmol) was solubilized in a 0.2 M NaHCO_3_ aqueous solution (50 mL) at room temperature under magnetic stirring for 10 min. Simultaneously, Boc_2_O (0.291 g, 1.33 mmol) was diluted with 1,4-dioxane (5 mL) and added dropwise over the PEG solution cooled to 0 °C using an ice bath (H_2_N-PEG_1500_-NH_2_:Boc_2_O molar ratio of 1:1). After the complete addition of Boc_2_O, the reaction mixture was allowed to magnetically stir for 20 h at room temperature. After the reaction was complete, the pH of the reaction mixture was adjusted to pH = 7.0 using HCl (1%), and the obtained solution was freeze-dried under reduced pressure, yielding a yellowish fluffy solid. The obtained solid was solubilized in anhydrous DMF and precipitated overnight from a cold ether in the refrigerator. The white precipitate was collected by centrifugation at 5000× *g* for 10 min, washed twice with cold ether, and dried in the oven under reduced pressure for 12 h, obtaining a white-yellowish precipitate yielding 957 mg (47%).

**^1^H-NMR** (400 MHz, CDCl_3_), δ (ppm): 5.01 (s, 1H, -N***H***-COO-), 3.62 (s, 130H, -(C***H***_2_-C***H***_2_-O)_n_), 3.54–3.51 (d, *J* = 6.2 Hz, 4H, -C***H***_2_-), 3.25–3.16 (dd, *J* = 11.2, 5.4 Hz, 2H, -C***H***_2_-), 2.81–2.74 (t, *J* = 6.7 Hz, 2H, -C***H***_2_-), 2.09 (bs, 2H, -N***H***_2_), 1.76–1.68 (m, 4H, -C***H***_2_-), and 1.41 (s, 9H, -C***H***_3_) (Figure S5). **^13^C-NMR** (100 MHz, CDCl_3_), δ (ppm): 156.02 (-NH-***C***OO-), 78.83 (-O-***C***(CH_3_)_3_), 72.54 (-(CH_2_-CH_2_-O)_n_-***C***H_2_-), 70.55 (-(***C***H_2_-***C***H_2_-O)_n_-), 61.63 (-***C***H_2_-O-(CH_2_-CH_2_-O)_n_-), 39.57 (-***C***H_2_-NH_2_), 38.54 (-***C***H_2_-NH-COO-), 33.25 (-***C***H_2_-CH_2_-NH_2_), 29.60 (-O-CH_2_-***C***H_2_-), and 28.45 (-O-C(***C***H_3_)_3_) (Figure S6). **ATR-FTIR** ν (cm^−1^): 3354 (N-H stretching), 2882 (C-H stretching), 1709 (C=O stretching), 1466 (C-H bending), 1340 (C-H bending), 1278 (C-N stretching), 1240 (C-N stretching), 1103 (C-O stretching), 947 (N-H wagging), 841 (N-H wagging), and 704 (C-H bending) (Figure S7). **Maldi-TOF MS/MS:** *m*/*z* calculated: 1596.99 (100%) and obtained: 1594.83 (Figure S8).

#### 2.3.3. Synthesis of the SQ-PEG_1500_-NH-Boc Amphiphilic Copolymer

The synthesis of the PEGylated squalene was accomplished using a slightly modified version of our previously reported protocol [58]. Briefly, in the first step, SQ-COOH (200 mg, 0.5 mmol) was solubilized in anhydrous DMF (3 mL), and the flask was flushed and kept under dried nitrogen. Next, under a nitrogen atmosphere, EDC-HCl (144 mg, 0.75 mmol) and NHS (86 mg, 0.75 mmol) were solubilized in anhydrous DMF (2 mL and 1 mL, respectively) and added dropwise over the SQ-COOH solution at room temperature under magnetic stirring. The reaction mixture was magnetically stirred for 4 h at room temperature under a nitrogen atmosphere. The reaction progress was monitored by TLC (5% MeOH in DCM). The in situ-obtained compound as an activated ester of squalenic acid (SQ-COONHS) was kept in DMF under a nitrogen atmosphere until further use. Next, SQ-COONHS (60 mg, 0.12 mmol) was diluted with anhydrous DMF (2 mL) under a nitrogen atmosphere, and TEA (19 mg, 0.19 mmol) was added to the obtained solution. Separately, H_2_N-PEG_1500_-NH-Boc (299 mg, 0.19 mmol) was solubilized in anhydrous DMF (4 mL) and added dropwise over the SQ-COONHS solution, and the reaction mixture was allowed to magnetically stir for 24 h at room temperature under a nitrogen atmosphere. After the reaction was completed, the mixture was diluted with dd-H_2_O and extracted with a mixture of DCM and n-hexane in a volume ratio of 1:1. The combined organic phases were washed twice with dd-H_2_O and once with brine, dried over anhydrous Na_2_SO_4_, and concentrated to dryness under reduced pressure using a rotary evaporator (10 mbar, 40 °C). The remaining residue, as an amorphous solid, was solubilized in 20 mL mQ-H_2_O and sonicated 3 times for 4 min at 120 W (sonication pulse was set at 2 s with 4 s between pulses), as described in the literature [59]. After sonication, the solution was filtered through an Amicon Ultra-15 (Merck, Darmstadt, Germany) filter unit with MWCO of 3000 Da at 5000× *g* for 60 min. The remaining residue was washed two times with mQ-water and filtered again through an Amicon Ultra-15 filter with an MWCO of 3000 Da at 5000× *g* for 60 min. Finally, the obtained solution was diluted with mQ-H_2_O and dialyzed against mQ-H_2_O for 48 h through a 3500 Da MWCO membrane (SnakeSkin from ThermoScientific, Waltham, MA, USA). The solution containing SQ-PEG_1500_-NH-Boc was collected from the dialysis tube and was freeze-dried under reduced pressure to obtain a white fluffy solid yielding 196.2 mg (80%).

**^1^H-NMR** (400 MHz, CDCl_3_), δ (ppm): 6.22–6.13 (bs, 1H, (PEG)-N***H***-CO-(SQ)), 5.21–5.07 (m, 5H, (SQ)-C***H***=), 4.98–4.88 (bs, 1H, (PEG)-N***H***-(COO)-*t*-bu), 3.65–3.60 (bs, 130H, (PEG)-C***H***_2_-C***H***_2_-O-), 3.59–3.56 (m, 4H, (PEG)-C***H***_2_-), 3.55–3.49 (dd, *J* = 11.8, 5.9 Hz, 4H, (PEG)-C***H***_2_-), 3.36–3.29 (dd, *J* = 12.3, 6.1 Hz, 2H, (PEG)-C***H***_2_-), 2.21–1.93 (m, 20H, (SQ)-C***H***_2_-), 1.77–1.71 (dd, *J* = 10.9, 6.1 Hz, 2H, (PEG)-C***H***_2_-), 1.66 (s, 3H, (SQ)-C***H***_3_), 1.59 (d, *J* = 6.1 Hz, 15H, (SQ)-C***H***_3_), and 1.42 (s, 9H, -*t*-bu-(C***H***_3_)_3_) (Figure S9). **^13^C-NMR** (100 MHz, CDCl_3_), δ (ppm): 172.92 (-***C***(O)-NH-), 172.90 (-O-***C***(O)-NH-), 135.14 (-***C***(CH_3_)=), 134.96 (-***C***(CH_3_)=), 134.90 (-***C***(CH_3_)=), 133.72 (-***C***(CH_3_)=), 131.24 (H_3_C-***C***(CH_3_)=), 125.06 (-***C***H=), 124.99 (-***C***H=), 124.39 (-***C***H=), 124.35 (-***C***H=), 124.24 (-***C***H=), 72.80 (-(CH_2_-CH_2_-O)_n_-***C***H_2_-), 70.50 (-(***C***H_2_-***C***H_2_-O)_n_-), 70.17 (-O-***C***(CH_3_)_3_), 61.58 (-***C***H_2_-O-(CH_2_-CH_2_-O)_n_-), 39.73 (-***C***H_2_-), 39.71 (-***C***H_2_-NH-), 39.64 (-***C***H_2_-NH-COO-), 35.44 (-***C***H_2_-CH_2_-NH_2_), 28.94 (-O-CH_2_-***C***H_2_-), 28.45 (-O-C(***C***H_3_)_3_), 28.26 (-***C***H_2_-), 26.75 (-***C***H_2_-), 26.64 (-***C***H_2_-), 25.69 (-***C***H_2_-), 17.67 (-***C***H_3_), 16.04 (-***C***H_3_), 15.99 (-***C***H_3_), and 15.91 (-***C***H_3_) (Figure S10). **ATR-FTIR** ν (cm^−1^): 3360 (N-H stretching), 2884 (C-H stretching), 1659 (C=O stretching), 1466 (C-H bending), 1341 (C-H bending), 1279 (C-N stretching), 1240 (C-N stretching), 1099 (C-O stretching), and 947 (N-H wagging), 841 (N-H wagging) (Figure S11). **Maldi-TOF MS/MS:** *m*/*z* calculated: 1980.32 (100%) and obtained: 2056.23 (Figure S12).

#### 2.3.4. Preparation of SQ-PEG_1500_-NH-Boc Unloaded Micelles

Due to the self-assembly properties of squalene moiety and the amphiphilic nature of the SQ-PEG_1500_-NH-Boc copolymer, in aqueous media, the preparation of SQ-PEG_1500_-NH-Boc unloaded micelles was accomplished by the ultrasound-assisted method, using the protocol published elsewhere [31,60]. The obtained SQ-PEG_1500_-NH-Boc, a white fluffy solid, was solubilized in mQ-H_2_O/PBS at a desired concentration and magnetically stirred for 24 h at ambient temperature (23 °C). It is well known that PEGylated squalene in aqueous media at a concentration close to or higher than CMC is instantly self-assembled into nano-sized micellar architectures with a hydrophobic core surrounded by a hydrophilic shell [60,61]. Also, it is expected that even if the concentration of SQ-PEG_1500_-NH-Boc is less than CMC, the copolymer will be assembled into an intramolecular aggregate when the squalene is surrounded by a PEG moiety [62]. In this context, for a homogenous distribution of the micellar formation sizes, first, the micelles were deconstructed by continuous ultrasonication (10 min at 120 W) of the aqueous solution containing SQ-PEG_1500_-NH-Boc using a UP200St-G Ultrasonic System equipped with a UP200St-TD Probe-Type transducer from HIELSCHER Ultrasonics and, secondly, for the micelle’s reconstruction, the solution was allowed to stabilize for 30 min under magnetic stirring at ambient temperature (23 °C). The obtained aqueous solution containing the micelles of SQ-PEG_1500_-NH-Boc was kept at 2–4 °C for further experiments.

#### 2.3.5. Preparation of Drug-Loaded Self-Assembled Nanotherapeutics

Due to the nature of the drug (MTx—hydrophobic and Cyt—hydrophilic), the preparation of the drug-loaded nanotherapeutics was achieved using two different methods.

- Preparation of the SQ-PEG_1500_-NH-Boc/MTx-loaded nanotherapeutic

An MTx-loaded nanotherapeutic was prepared using an ultrasound-assisted dropping method, following a slightly modified protocol as described elsewhere [12]. Initially, 200 mg of an SQ-PEG_1500_-NH-Boc copolymer was dissolved in 40 mL of mQ-H_2_O. Concurrently, 15 mg of MTx was dispersed in 10 mL of THF with continuous ultrasonication. The resulting solution was slowly injected into 20 mL of mQ-H_2_O while maintaining continuous ultrasonication. In the next step, the THF was mostly evaporated, and the MTx dissolved in mQ-H_2_O was carefully dropwise injected into the SQ-PEG_1500_-NH-Boc aqueous solution, still under continuous ultrasonication. Subsequently, the obtained mixture underwent thermal treatment at 60 °C for 2 h with continuous stirring. Finally, the solution was allowed to reach the ambient temperature (23 °C) over a 24 h period. To remove any unloaded drug, a 24 h mQ-H_2_O dialysis was conducted using a 3500 Da MWCO membrane. The resulting MTx-loaded nanotherapeutic aqueous solution was collected from the dialysis bag and stored at 4 °C for further studies.

- Preparation of the SQ-PEG_1500_-NH-Boc/Cyt-loaded nanotherapeutic

The Cyt-loaded nanotherapeutic was synthesized using an ultrasound-assisted solvent evaporation method with a slightly modified protocol described elsewhere [12]. In summary, a mixture of mQ-H_2_O (18 mL) and THF (18 mL) was used to solubilize 90 mg of the SQ-PEG_1500_-NH-Boc copolymer under continuous ultrasonication. Simultaneously, the Cyt antitumoral drug was solubilized in a mixture of mQ-H_2_O (6 mL) and THF (6 mL) under continuous ultrasonication. The resulting solutions were combined and subjected to continuous sonication, followed by thermal treatment at 60 °C for 2 h with continuous stirring. The solution was then allowed to cool to ambient temperature (23 °C) over a 24 h period. Next, THF evaporation was performed, and the remaining aqueous solution was dialyzed against mQ-H_2_O for 24 h using a 3500 Da MWCO membrane. Finally, the Cyt-loaded nanotherapeutic was collected from the dialysis bag as an aqueous solution and stored at 4 °C until further studies.

## 3. Results and Discussion

### 3.1. Synthesis and Characterization of the SQ-PEG_1500_-NH-Boc Copolymer

**Scheme 1:**
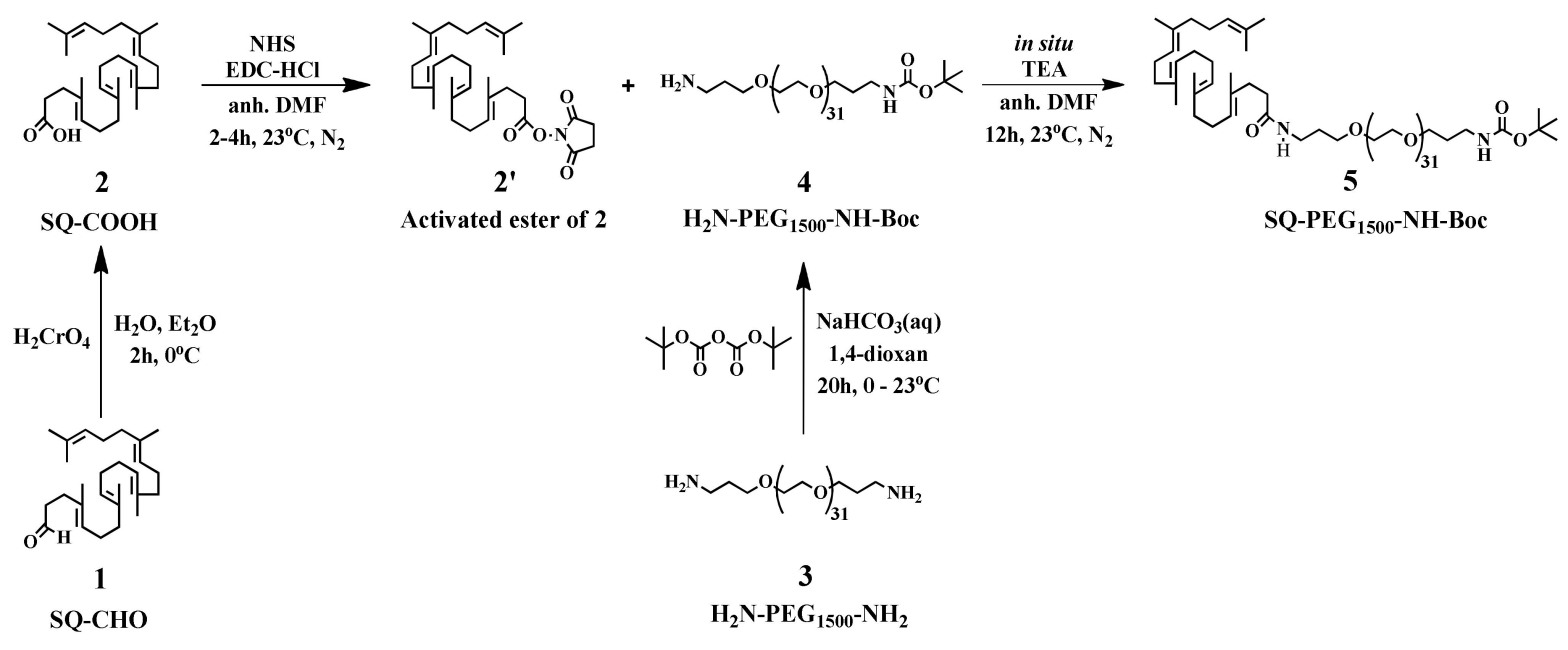
Synthesis pathway of the SQ-PEG_1500_-NH-Boc copolymer.

The synthesis of the SQ-PEG_1500_-NH-Boc copolymer (**5**) was achieved in a three-step reaction pathway using slightly modified protocols described in the literature (Scheme 1) [58,63]. In the first step, SQ-COOH (**2**) was obtained by a Jones oxidation of the squalene aldehyde (**1**) [63], which was previously obtained from commercially available squalene using protocols reported in the literature [31,54,55,56]. In the next step, using the protocol described by Wang et al. [57], the H_2_N-PEG_1500_-NH_2_ (**3**) was subjected to a Boc protection of the amine group at one end to avoid an uncontrolled reaction of both amine groups. In the final step, in a one-pot reaction, the corresponding active ester (**2′**) of the previously obtained SQ-COOH was obtained by treatment with EDC-HCl and NHS coupled by a nucleophilic attack of the primary amine group from the H_2_N-PEG_1500_-NH-Boc (**4**) to obtain the SQ-PEG_1500_-NH-Boc copolymer (**5**).

**Figure 1:**
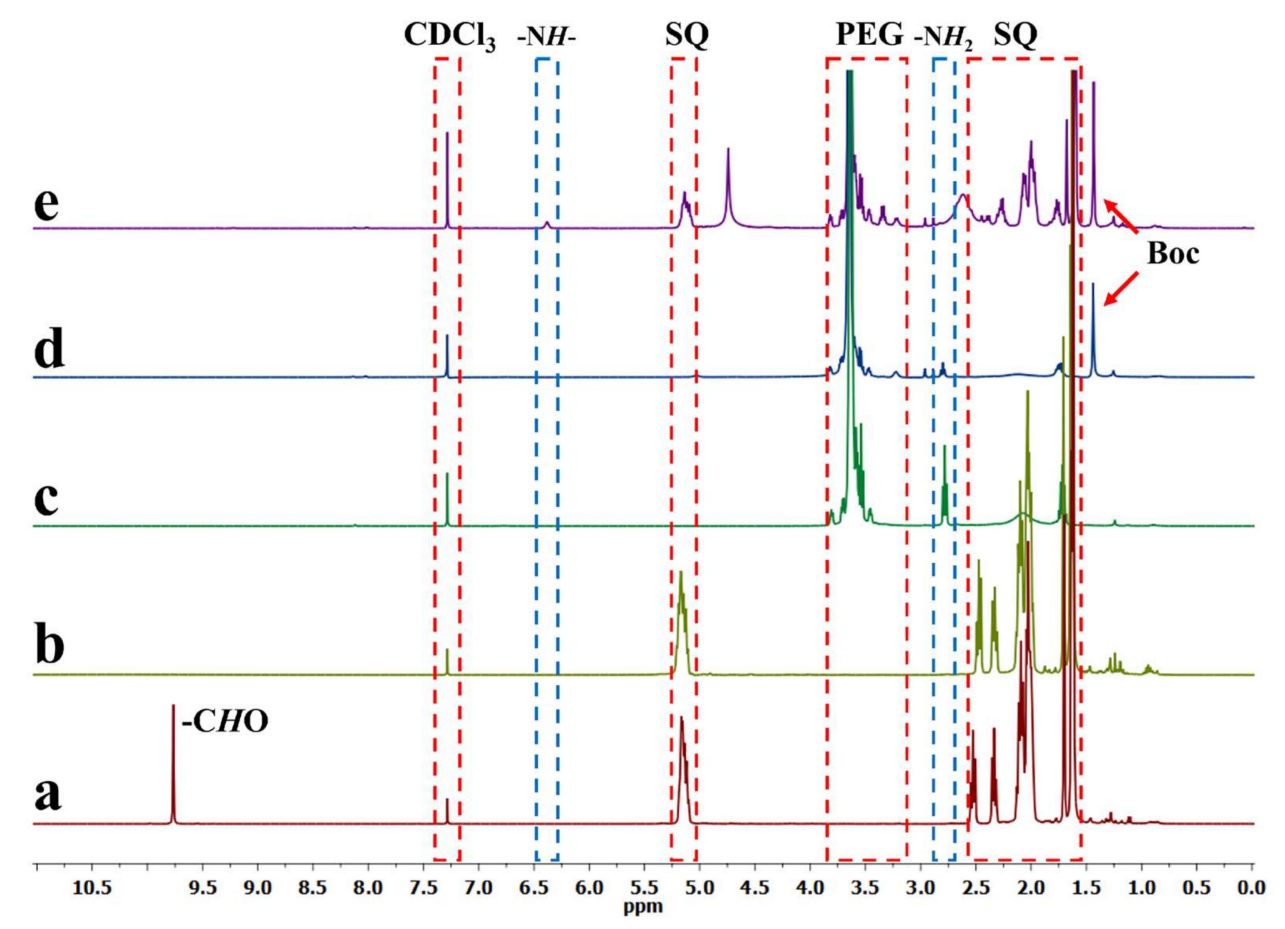
Overlapping ^1^H-NMR (CDCl_3_, 400 MHz) spectra of (**a**) SQ-CHO; (**b**) SQ-COOH; (**c**) H_2_N-PEG_1500_-NH_2_; (**d**) H_2_N-PEG_1500_-NH-Boc; (**e**) SQ-PEG_1500_-NH-Boc.

**Figure 2:**
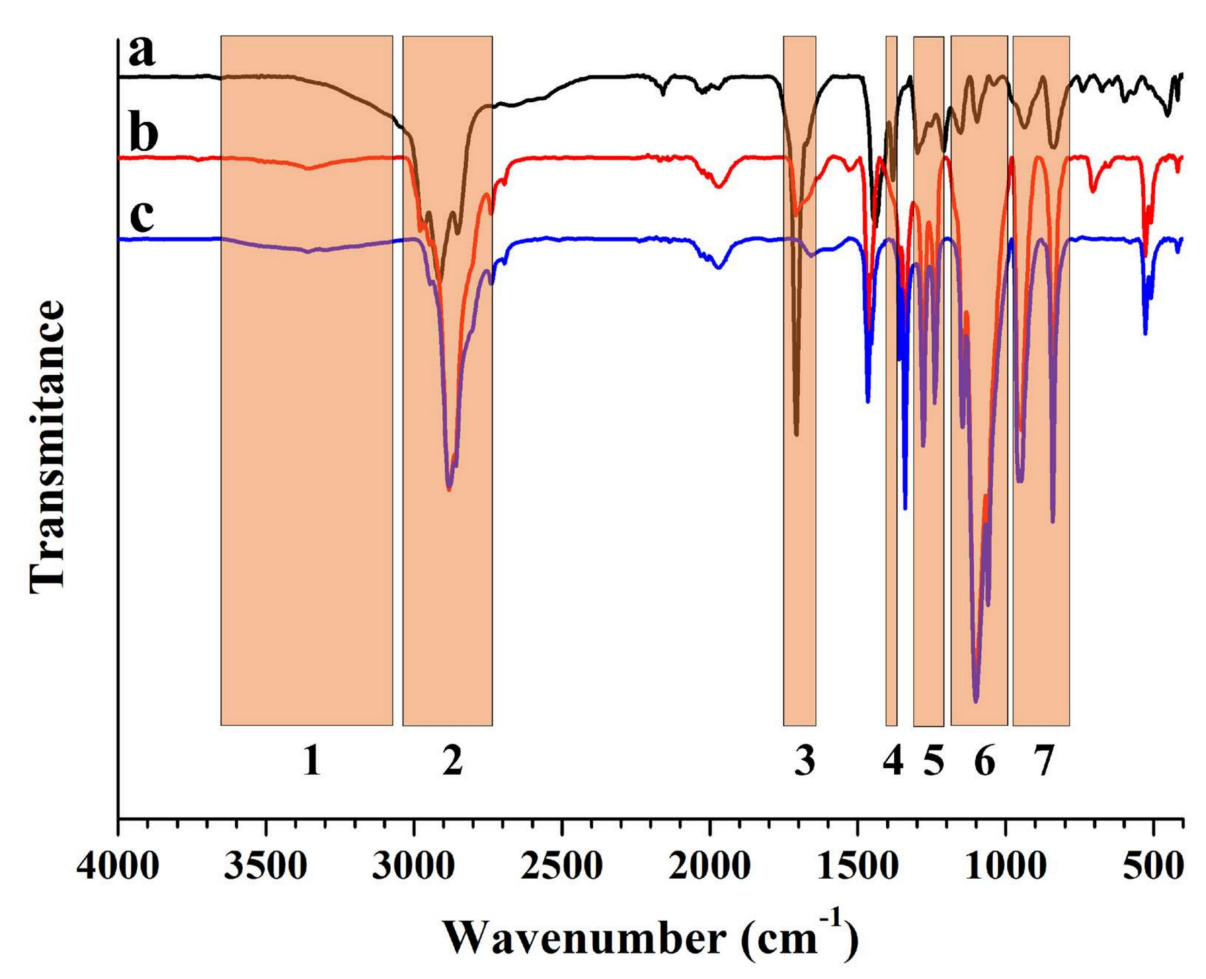
Overlapping ATR-FTIR spectra of (**a**) SQ-COOH; (**b**) H_2_N-PEG_1500_-NH-Boc; (**c**) SQ-PEG_1500_-NH-Boc. Where **1**: νN-H; **2**: νC-H (alkyl); **3**: νC=O; **4**: νC-O (carboxyl); **5**: νC-N; **6**: νC-O (ether); **7**: overlapping of νO-H and νN-H.

Successful syntheses of the intermediary compounds and the final SQ-PEG_1500_-NH-Boc copolymer were confirmed by proton and carbon NMR, ATR-FTIR, and Maldi-TOF, as shown in Figures S1–S12 in the Supplementary Materials, and the overlapping of the ^1^H-NMR and ATR-FTIR spectra are represented in Figure 1 and Figure 2, respectively, for better visualization.

In Figure 1, the typical chemical shifts of the protons from the squalene structure were found at δ = 5.22–5.06 ppm assigned to methylene protons and 2.50–1.60 ppm assigned to alkyl protons with slight modifications in the case of the final product of SQ-PEG_1500_-NH-Boc. Moreover, during the synthesis process, the disappearance of specific signals and the appearance of new ones was observed. For example, the NMR signal of the carbonylic proton from the starting material (SQ-CHO) at 9.75 ppm disappeared in the first synthesis step when the carboxylic group was formed. In the case of amine protection from the PEG with Boc, the partial disappearance of primary amine protons from δ = 2.09 ppm and the appearance of new NMR signals at δ = 5.00 ppm (specific for secondary amine protons) and δ = 1.40 ppm (specific for alkyl protons from the Boc protective group) could be observed. The structure of the final product was confirmed by the appearance of the amide proton at δ = 6.36 ppm and the remaining other typical NMR signals with slight modification due to some intramolecular interactions.

The ATR-FTIR spectra of SQ-COOH, H_2_N-PEG_1500_-NH-Boc, and SQ-PEG_1500_-NH-Boc were represented in Figure 2, and specific modifications during the synthesis process were highlighted. The weak bands at 3400 cm^−1^ were attributed to N-H stretching vibrations in the case of H_2_N-PEG_1500_-NH-Boc and SQ-PEG_1500_-NH-Boc FTIR spectra; these bands are accompanied by the bands at 900–800 cm^−1^, which are attributed to N-H wagging vibrations of primary and secondary amine groups. The strong bands at 2900–2800 cm^−1^ were assigned to the C-H stretching vibration of alkyl groups in the studied compounds. In the case of SQ-COOH, the characteristic bands at 1700 and 1400 cm^−1^ are attributed to asymmetric and symmetric stretching vibrations of the -COO functional group, and these bands are not present in the other two FTIR spectra that belong to H_2_N-PEG_1500_-NH-Boc and SQ-PEG_1500_-NH-Boc derivatives. Similarly, the strong characteristic bands at 1100 cm^−1^ were assigned to stretching vibrations of C-O from the ether group that belong to the ethylene glycol repetitive unit, and the strong bands at 1279 and 1240 cm^−1^ were attributed to C-N stretching vibrations. These bands were not present in the SQ-COOH FTIR spectrum. The bands between 950 and 800 cm^−1^ were assigned to overlap C-H and N-H wagging vibrations.

### 3.2. Self-Assembly Behavior of the SQ-PEG_1500_-NH-Boc Amphiphilic Copolymer

**Figure 3:**
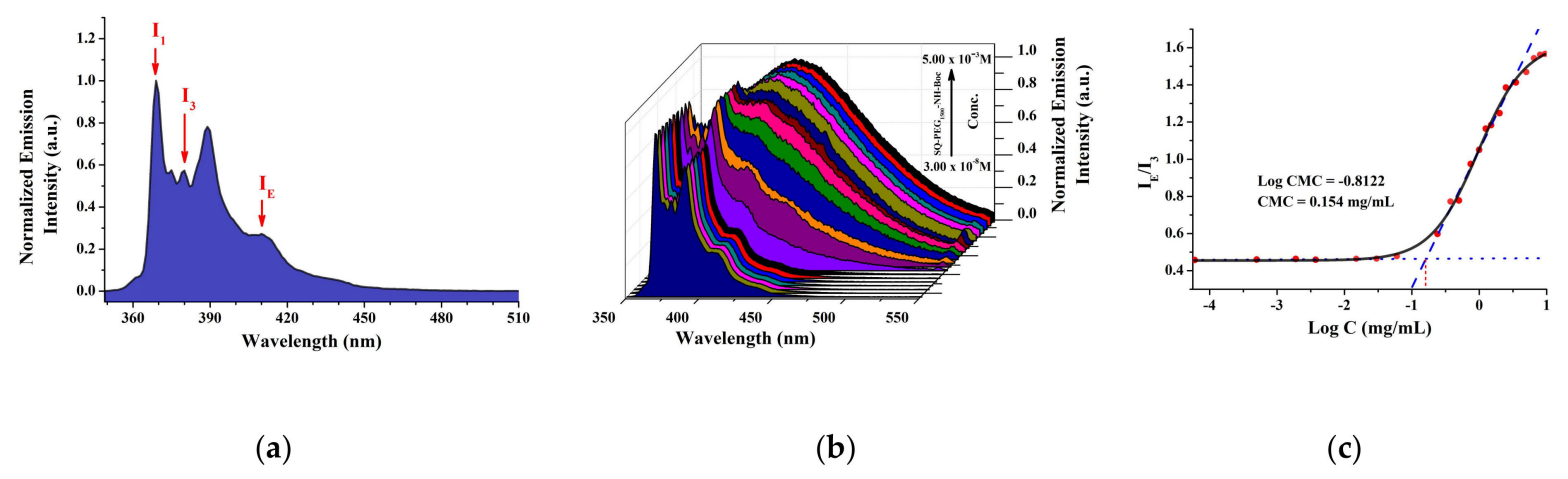
CMC study of SQ-PEG_1500_-NH-Boc in PBS solutions with a pH value of 7.4 in the presence of pyrene using the spectrofluorimetric method. (**a**) Normalized emission spectrum of pyrene in an aqueous solution; (**b**) normalized emission spectra of pyrene in the presence of different concentrations of SQ-PEG_1500_-NH-Boc (ranged between 3 × 10^−8^ M and 5 × 10^−3^ M), λ_ex_ = 334 nm; (**c**) Boltzmann sigmoidal fitting of I_E_/I_3_ as a function of logC (mg/mL, concentration of SQ-PEG_1500_-NH-Boc).

The self-assembly behaviour of the SQ-PEG_1500_-NH-Boc amphiphilic copolymer in an aqueous medium was first evaluated by recording the fluorescence spectra of pyrene solutions in water at a constant concentration (10^−7^M) in the presence of SQ-PEG_1500_-NH-Boc at various concentrations (Figure 3). The CMC value was obtained by plotting the curve I_E_/I_3_ versus log(c), where **I_E_** is excimer intensity, **I_3_** is the intensity of the third emission maximum, and **c** is the concentration of SQ-PEG_1500_-NH-Boc (mg/mL), as described in our previous work [31]. It is generally known that pyrene in water solutions presents five distinct emission peaks in the 360–400 nm region (Figure 3a). As previously stated, the intensity ratio of the first and third emission maxima (I_1_/I_3_) in the pyrene fluorescence spectra provides information regarding the local polarity of the probe’s surroundings; higher or lower values of this ratio are related to polar or non-polar environments of the probe [62]. Also, the ratio between excimer intensity (the excited state of the pyrene dimer at around 410 nm) and the third emission maximum (I_E_/I_3_) is another important parameter that provides information on the polarity of the pyrene surrounds [64].

In this study, the accurate CMC value of the SQ-PEG_1500_-NH-Boc in a PBS solution at a pH value of 7.4 was obtained by calculating I_E_/I_3_ from pyrene fluorescence emission spectra registered as fluorescence intensity vs. SQ-PEG_1500_-NH-Boc concentration at excitation wavelengths of 334 nm and a pyrene concentration of 10^−7^ M, as previously described by us [31,62]. Figure 3b shows the normalized emission spectra of pyrene aqueous solutions at 10^−7^M with different concentrations in an SQ-PEG_1500_-NH-Boc copolymer. When the SQ-PEG_1500_-NH-Boc concentration is increased, the intensities of pyrene unimers (λ_em_ = 370 to 390 nm) and excimers (λ_em_ = 410 nm) also increase. Boltzmann sigmoidal fitting was used to depict the I_E_/I_3_ as a function of logC (mg/mL, SQ-PEG_1500_-NH-Boc) and demonstrate a non-linear increase in I_E_/I_3_ values with increasing an SQ-PEG_1500_-NH-Boc concentration (Figure 3c). Additionally, it should be highlighted that the increase in I_E_/I_3_ values is caused by the amplified excimer intensity, indicating that pyrene is surrounded by the hydrophobic region of SQ-PEG_1500_-NH-Boc during the formation of micellar entities. The intersection of the two tangents from the Boltzmann sigmoidal fitting of I_E_/I_3_ as a function of logC led to obtaining a CMC value of 0.154 mg/mL for SQ-PEG_1500_-NH-Boc in a PBS solution with a pH value of 7.4 (Figure 3c). The CMC value is similar to the CMC value of PEGylated squalene, as determined in our previous work [31].

### 3.3. Preparation and Characterization of SQ-PEG_1500_-NH-Boc Micelles

**Scheme 2:**
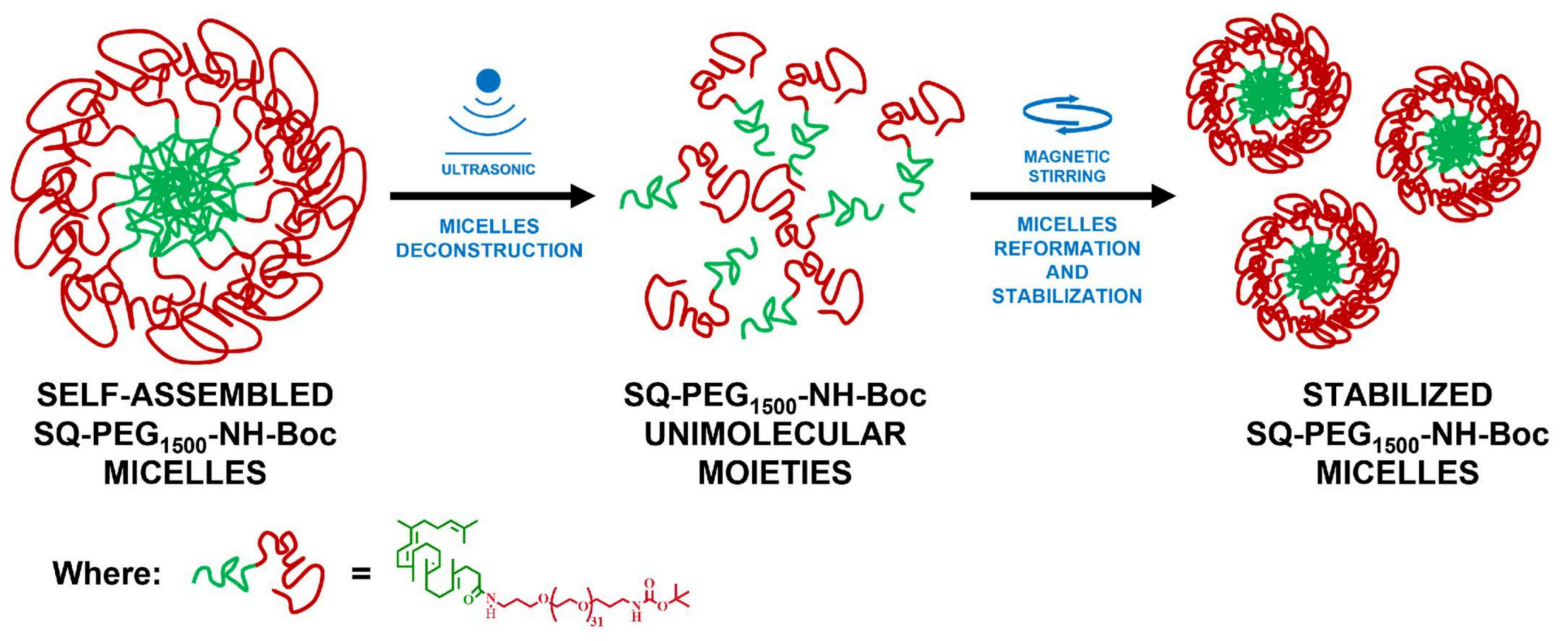
Schematic representation of SQ-PEG_1500_-NH-Boc micelles during the deconstruction-reformation process (green is squalene moiety and red is PEG).

Naturally, SQ-PEG_1500_-NH-Boc micelles are formed instantly when the copolymer is solubilized in aqueous solutions with concentrations close to or higher than the CMC value [62]. At a concentration below CMC, SQ-PEG_1500_-NH-Boc micelles disassemble into free molecular unimers when the squalene is surrounded by a PEG moiety [31]. For a uniform dispersion, the formed micelles are first deconstructed, and subsequently reformed in a PBS solution by the commonly used ultrasonic method, as described in the Materials and Methods section and shown in Scheme 2.

Briefly, SQ-PEG_1500_-NH-Boc micelles in PBS solutions were subjected to ultrasonic treatment for a couple of minutes to deconstruct SQ-PEG_1500_-NH-Boc micelles in unimolecular moieties, allowing the rearrangement of the uniformly dispersed SQ-PEG_1500_-NH-Boc micelles under magnetic stirring at room temperature for 30 min. After the ultrasonic process, the obtained PBS solutions containing SQ-PEG_1500_-NH-Boc micelles at desired concentrations were kept at 2–4 °C and further analysed within 48 h to avoid aggregation and destabilization of the systems.

#### 3.3.1. DLS Analysis in PBS Solutions

**Figure 4:**
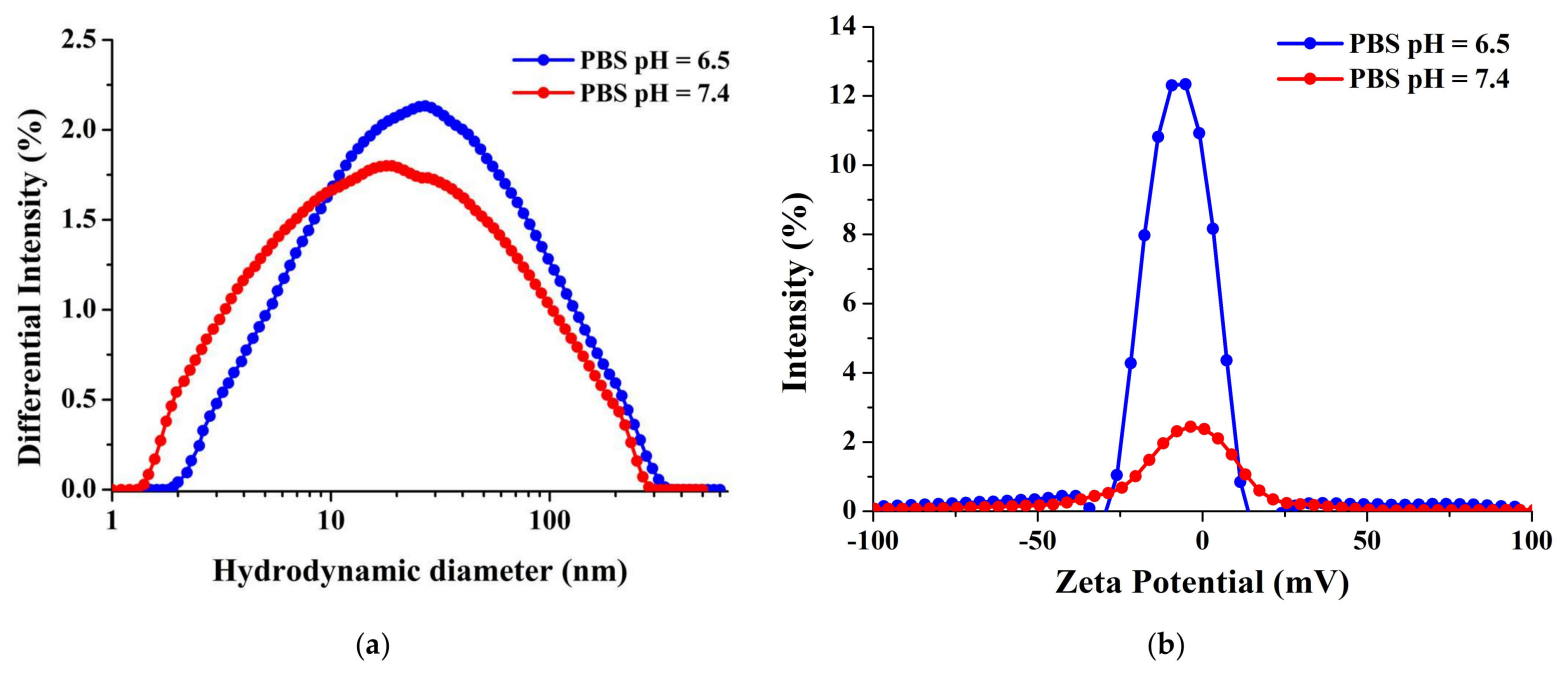
Colloidal features of SQ-PEG_1500_-NH-Boc micelles in PBS with a pH of 6.5 and 7.4: (**a**) hydrodynamic diameter distributions; (**b**) average zeta potential plots. The mass concentration of the SQ-PEG_1500_-NH-Boc copolymer was 1.25 mg/mL at 23 °C.

The behaviour of SQ-PEG_1500_-NH-Boc in PBS solutions with concentrations above the CMC value of 0.154 mg/mL with different pH levels (6.5 or 7.4) was investigated in terms of particle size and zeta potential using the DLS technique (Figure 4). The data obtained from these experiments are summarized in Table 1. The results from the DLS study indicated that the colloidal properties of SQ-PEG_1500_-NH-Boc micellar nano-constructs were influenced by the pH of the PBS solution. As shown in Figure 4a and Table 1, at both pH values, the SQ-PEG_1500_-NH-Boc micelles exhibited unimodality and wide H_d_ distributions (PDI = 0.252 ± 0.017 at pH 7.4 and PDI = 0.440 ± 0.026 at pH 6.5), with average values that were affected by the pH. At the physiological pH of 7.4, the mean particle size was found to be 36.4 ± 2.6 nm, which was smaller compared to the 44.6 ± 4.4 nm obtained at the tumoral pH value of 6.5 (Table 1). This observation suggests that the SQ-PEG_1500_-NH-Boc micellar structure undergoes partial deconstruction at lower pH values, indicating a pH-responsive characteristic that can facilitate the targeted release of encapsulated drugs, which is consistent with findings from other published studies [65,66].

The colloidal stability and the surface charge of the SQ-PEG_1500_-NH-Boc micelles in PBS solutions were also described by the obtained zeta potential values (Figure 4b). From the obtained results (Table 1), it could be observed that the SQ-PEG_1500_-NH-Boc micelles have negatively charged surfaces due to negative values of the zeta potential, which can be attributed to the presence of the -PEG_1500_-NH-Boc moieties at the surface of the nano-constructs. Additionally, it was found that the SQ-PEG_1500_-NH-Boc micelles’ colloidal stability was lower in physiological pH than tumoral pH. This phenomenon may be caused by the fact that micellar architectures are packed more tightly at a pH of 7.4 due to physical interactions between squalene molecules and PEG moieties, which could reduce the amount of PEG moieties on the surface of the micelles and cause fewer interactions with the aqueous medium The colloidal stability at both pH values is acceptable due to the zeta potential values being between +10 and −10 mV [67].

Further analysis of the SQ-PEG_1500_-NH-Boc solutions using the DLS technique was achieved by analysing the copolymer at three concentrations to observe if its colloidal features were concentration-dependent. For this study, the concentration of an SQ-PEG_1500_-NH-Boc copolymer in a PBS solution was above CMC due to the equipment limitations and varied from 0.75 mg/mL to 1.25 mg/mL with an intermediary concentration of 1.00 mg/mL, and the results are presented in the Supplementary Materials (Figure S13 and Table S1). From the results, we can observe that the colloidal features of the SQ-PEG_1500_-NH-Boc copolymer are dependent on the concentration. In PBS with a pH value of 6.5, the hydrodynamic size of SQ-PEG_1500_-NH-Boc micelles increases when decreasing the concentration, while in PBS 7.4, the hydrodynamic sizes decrease when decreasing the concentration. The colloidal stability of the SQ-PEG_1500_-NH-Boc micelles in the PBS solution, determined by measuring zeta potentials, increases with a decrease in concentration at both pH values, and the zeta potential values are negative in both studied cases.

#### 3.3.2. STEM Analysis

**Figure 5:**
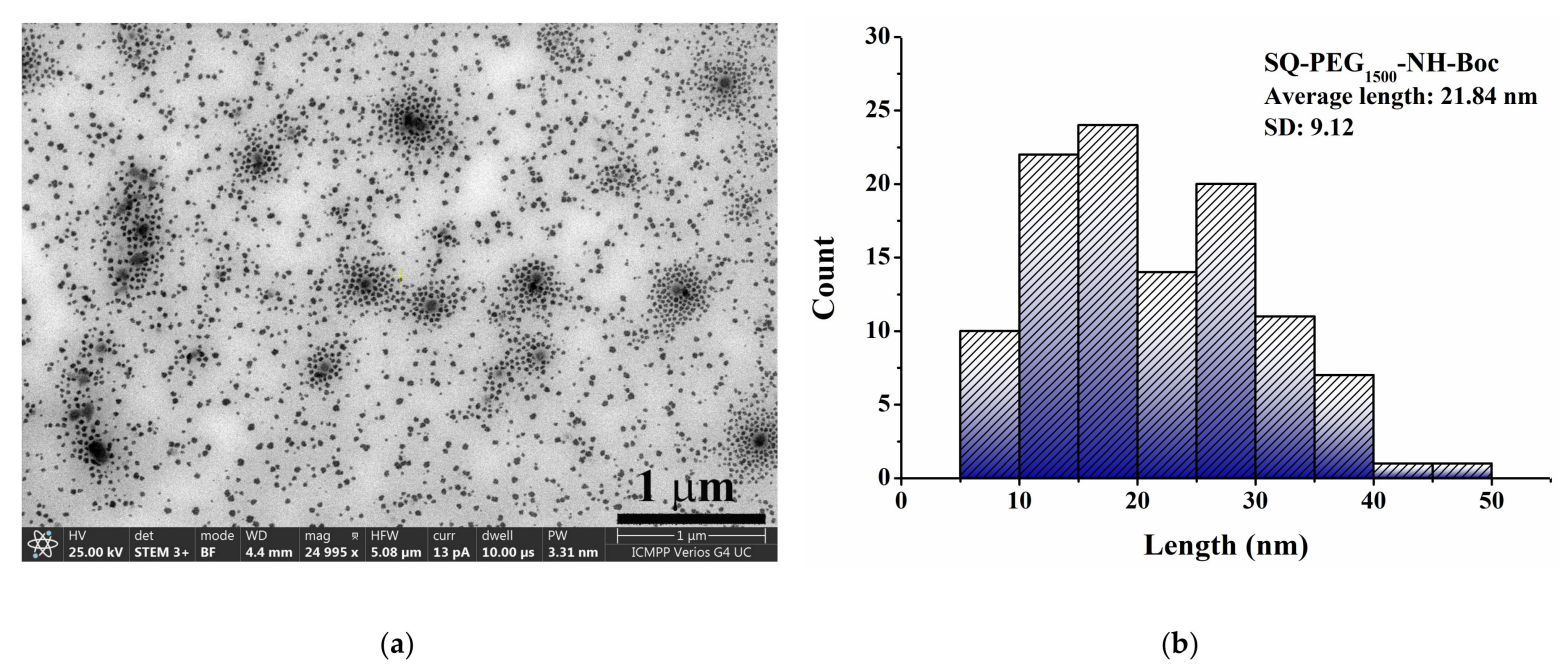
STEM morphological analysis of SQ-PEG_1500_-NH-Boc: (**a**) STEM image of SQ-PEG_1500_-NH-Boc micelles at 1 µm scale; (**b**) size distributions plot of SQ-PEG_1500_-NH-Boc micelles (*n* = 120 micelles).

The morphological features of the SQ-PEG_1500_-NH-Boc micelles in a static state were evaluated using the STEM technique (Figure 5). STEM analysis revealed that the SQ-PEG_1500_-NH-Boc micelles had “core-shell” spherical morphologies with nanometric dimensions that aggregate into bigger clusters as concentration increases (Figure 5a). The size distribution plot of SQ-PEG_1500_-NH-Boc micelles (Figure 5b), which was obtained by measuring 120 individual entities in Figure 5a using ImageJ software v1.54d [47], presented a narrow size distribution with dimensions ranging from 5 to 50 nm with a mean value of 21.84 ± 9.12 nm. The micelle sizes obtained from STEM were found to be smaller than those obtained by DLS measurement at both pH values (36.4 ± 2.6 nm and 44.6 ± 4.4 nm respectively), as shown in Figure 4a.

According to the literature [67,68,69], the difference between the SQ-PEG_1500_-NH-Boc micelles’ sizes that were obtained via the STEM and DLS technique can be explained as the DLS technique measures the solvated state where there are solvent molecules associated with the SQ-PEG_1500_-NH-Boc micelles (hydrodynamic radius), whereas STEM analysis observes the SQ-PEG_1500_-NH-Boc micelles in a dry state.

### 3.4. Preparation of Drug-Loaded SQ-PEG_1500_-NH-Boc Micelles

**Scheme 3:**
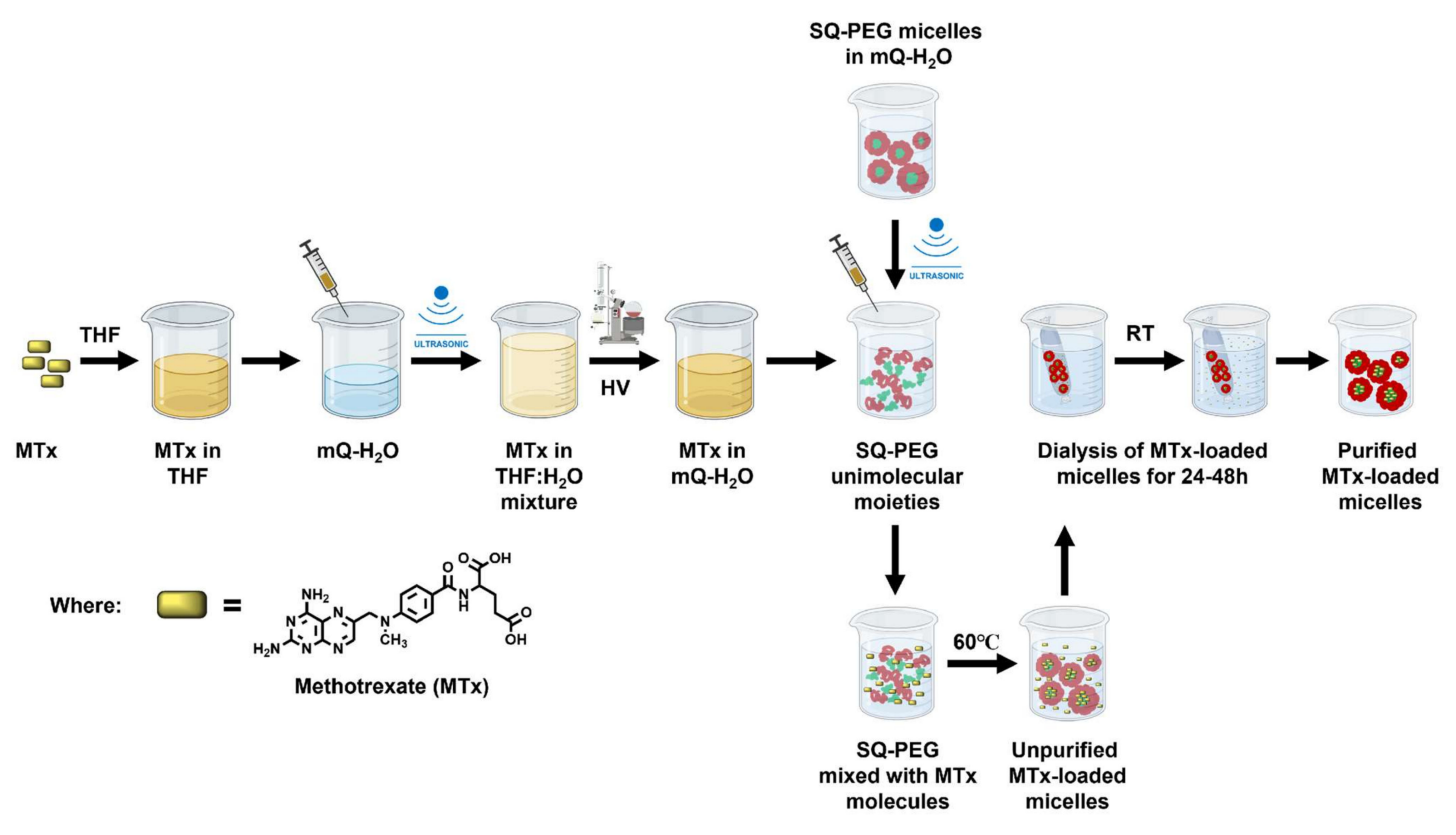
Schematic representation of the experimental procedure for obtaining the MTx-loaded micelles in an aqueous solution by the nanoprecipitation method. Where SQ-PEG = SQ-PEG_1500_-NH-Boc.

**Scheme 4:**
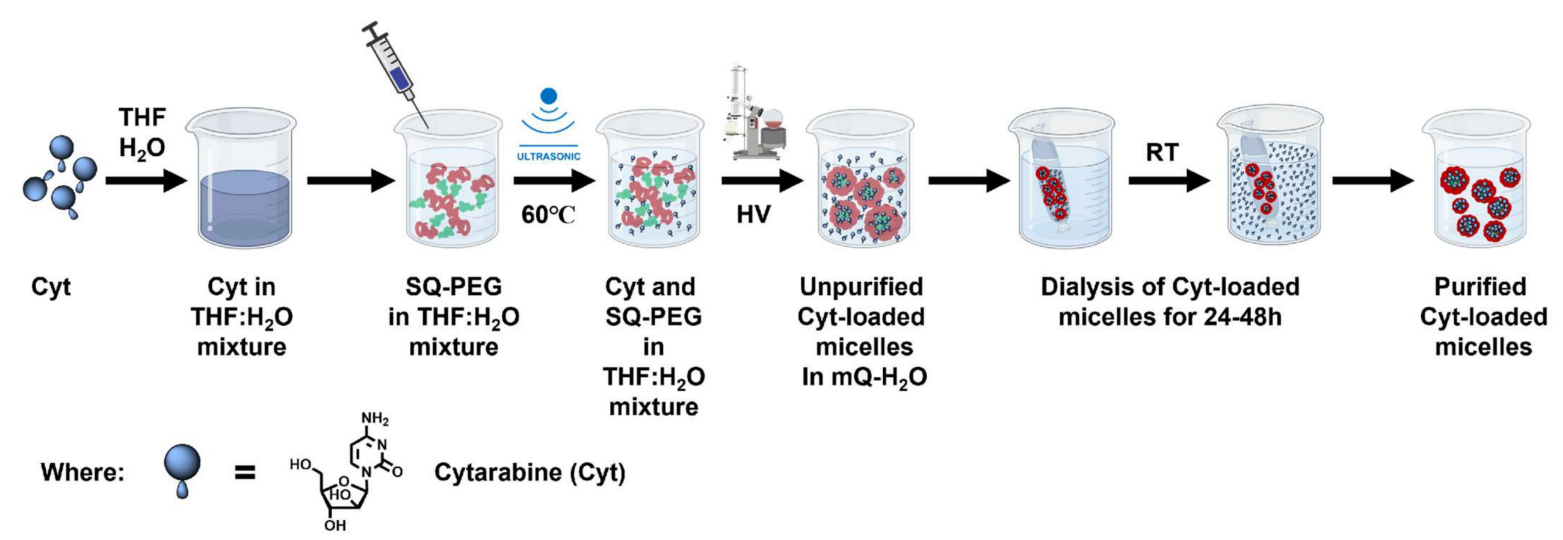
Schematic representation of the experimental procedure for obtaining the Cyt-loaded micelles in aqueous solution by the nanoprecipitation method. Where SQ-PEG = SQ-PEG_1500_-NH-Boc.

The drug-loaded SQ-PEG_1500_-NH-Boc micelles with methotrexate and cytarabine were obtained using the optimized version of the published experimental procedures [12,24] and are explained in detail in the Section 2. Briefly, due to the nature of the drug (MTx—hydrophobic and Cyt—hydrophilic), the preparation of the drug-loaded nanotherapeutics was achieved using two different methods (Scheme 3 and Scheme 4).

#### 3.4.1. Preparation of MTx-Loaded SQ-PEG_1500_-NH-Boc Micelles

The MTx (an antitumoral drug) was encapsulated (Scheme 3) using the nanoprecipitation method by solubilizing the drug in THF, and the obtained solution was injected in ultrapure water under continuous sonication. The solvent was removed using reduced pressure, and the MTx aqueous solution was further injected into an aqueous solution containing the SQ-PEG_1500_-NH-Boc micelles (with SQ-PEG_1500_-NH-Boc concentration above a CMC value of 0.154) under continuous sonication to allow the SQ-PEG_1500_-NH-Boc micelles to deconstruct and encapsulate the MTx during reformation. The obtained solution was stabilized by applying a thermal treatment at 60 °C for 2 h and was allowed to reach ambient temperature (23 °C) overnight under darkness. Finally, the unloaded MTx was removed by the commonly used dialysis method in ultrapure water for 24–48 h.

#### 3.4.2. Preparation of Cyt-Loaded SQ-PEG_1500_-NH-Boc Micelles

Cyt (a therapeutic drug) was encapsulated (Scheme 4) using the solvent evaporation method by solubilizing the drug into a THF-water mixture, and the obtained solution was added over a THF-water solution containing the SQ-PEG_1500_-NH-Boc copolymer. The obtained mixture was continuously sonicated for a couple of minutes and subjected to a thermal treatment at 60 °C for 2 h. After reaching ambient temperature (23 °C), the THF was removed under reduced pressure, and the remaining aqueous solution containing unloaded and encapsulated Cyt in SQ-PEG_1500_-NH-Boc micelles was subjected to a dialysis procedure against ultrapure water for 24–48 h to remove the unloaded Cyt.

### 3.5. Characterization of Drug-Loaded SQ-PEG_1500_-NH-Boc Micelles

#### 3.5.1. Quantification of Drug Encapsulation Efficiency and Loading Capacity

**Figure 6:**
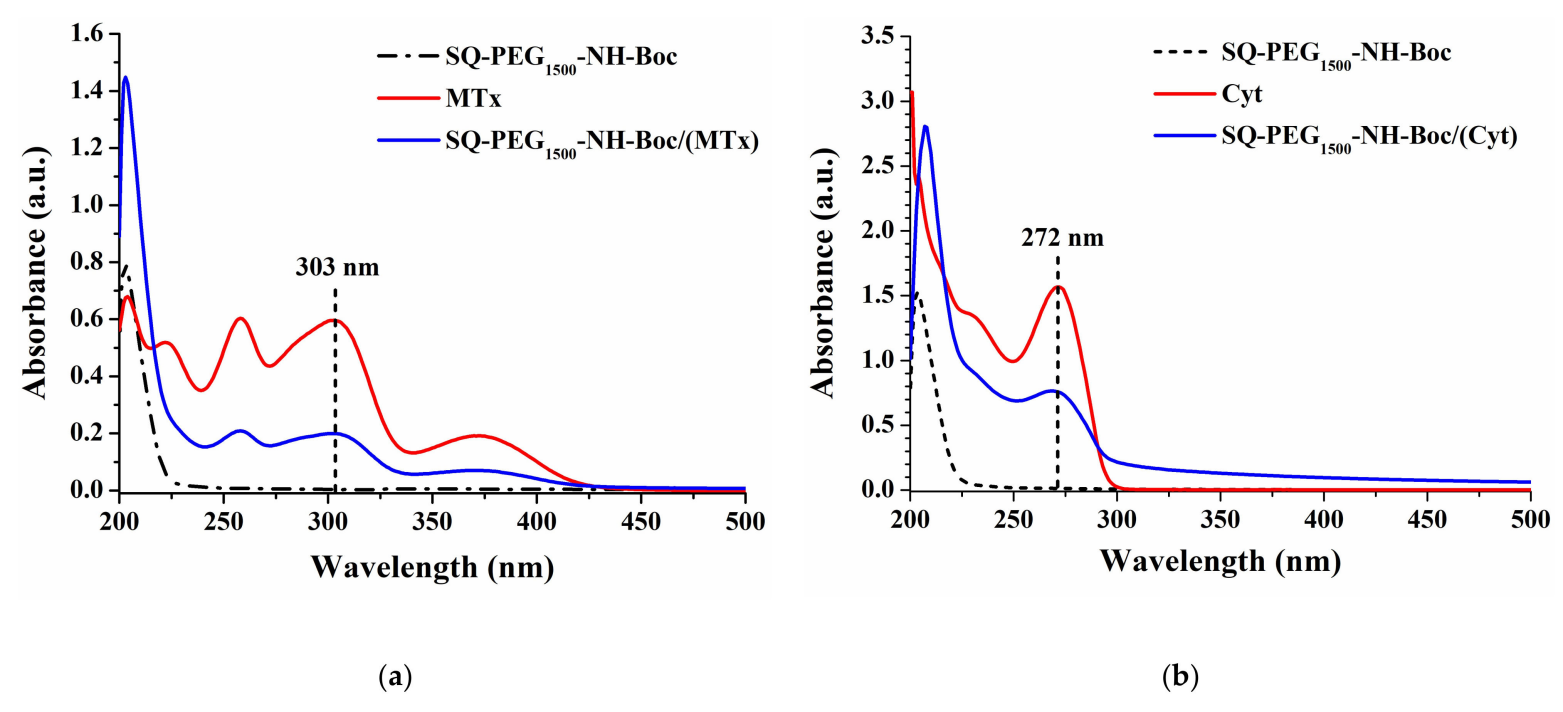
Overlapping the absorbance spectra recorded in the PBS solutions with a pH value of 7.4: (**a**) absorbance spectra of unloaded SQ-PEG_1500_-NH-Boc micelles, free MTx and MTx-loaded SQ-PEG_1500_-NH-Boc micelles; (**b**) absorbance spectra of unloaded SQ-PEG_1500_-NH-Boc micelles, free Cyt and Cyt-loaded SQ-PEG_1500_-NH-Boc micelles.

The encapsulation efficiencies (%EE) and drug loading capacities (%DL) of the MTx and Cyt drugs loaded into SQ-PEG_1500_-NH-Boc micelles were determined using the UV-vis spectroscopy method via calibration curves for each studied drug (MTx and Cyt) (Figure 6 and Figures S14 and S15) [70]. Briefly, after obtaining the drug-loaded SQ-PEG_1500_-NH-Boc micelles in triplicate, the PBS solutions with pH 7.4 were subjected to UV-Vis analysis, monitoring the characteristic absorbance maxima of the drugs (303 nm for MTx and 272 nm for Cyt). Using the line equations from calibration curves and the equations from the Materials and Methods section, we obtained the average values ± SD of (%EE) and (%DL), which are presented in Table 2.

The obtained data show that the SQ-PEG_1500_-NH-Boc micelles have a similar drug loading capacity for both studied drugs, MTx and Cyt, with %DL values of 3.99 ± 0.086 and 3.48 ± 1.715, respectively, although the drugs are different in terms of hydrophilic/hydrophobic properties. Regarding the encapsulation efficiency values, it could be observed that the obtained values revealed a poor encapsulation efficiency in the case of the hydrophilic drug (Cyt) with a value of 44.60 ± 6.862%EE compared with the encapsulation efficiency of the hydrophobic drug (MTx), which has a value of 54.02 ± 0.303%EE.

The difference between the obtained %EE values could be explained by the self-assembly of the SQ-PEG_1500_-NH-Boc micelles in spherical entities with a hydrophobic core surrounded by a hydrophilic shell, which allows them to interact with the studied drugs, depending on their hydrophobic/hydrophilic property. The hydrophobic drug will be entrapped into the hydrophobic region of the micellar architectures, whereas the hydrophilic drug will interact more with the hydrophilic shell, and a small amount of the drug will be found inside the core of the micelle.

#### 3.5.2. Quantification of Drug Encapsulation Efficiency and Loading Capacity

**Figure 7:**
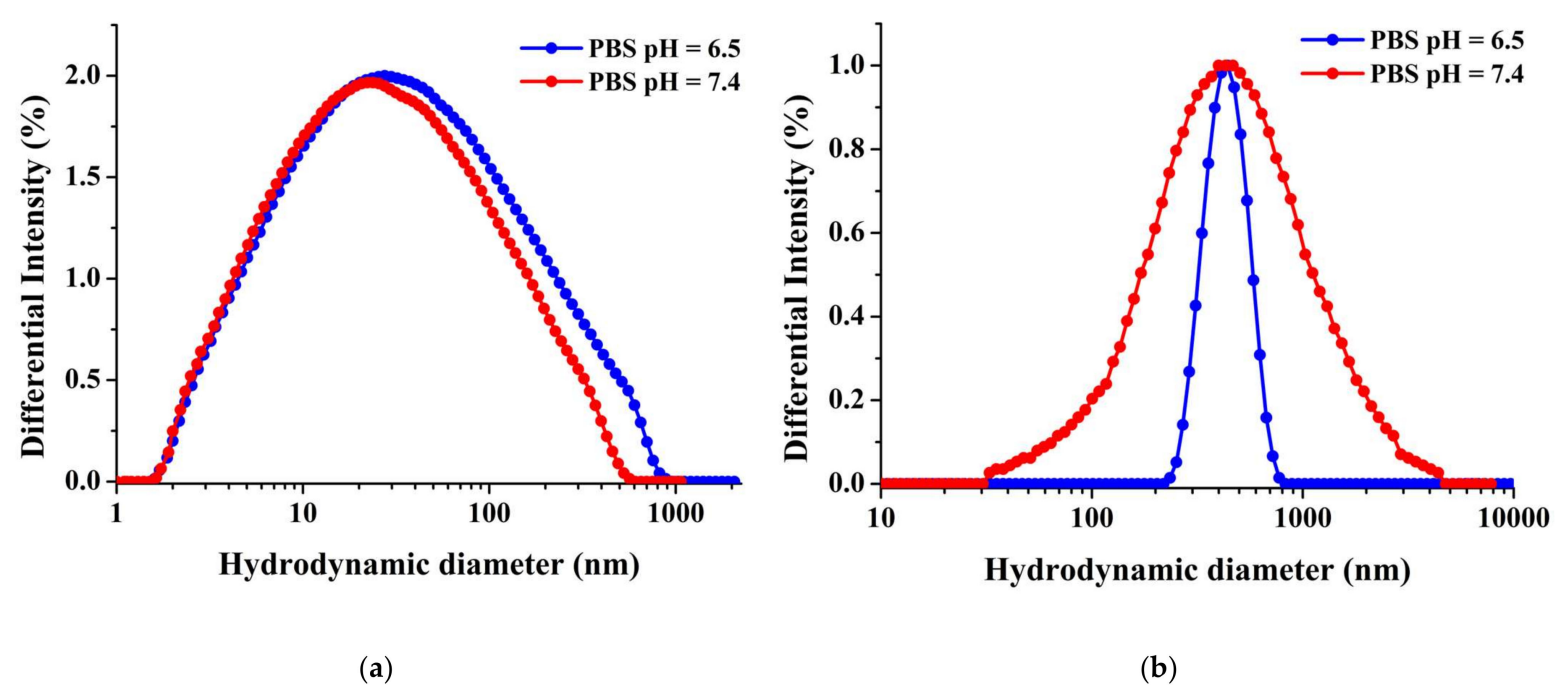

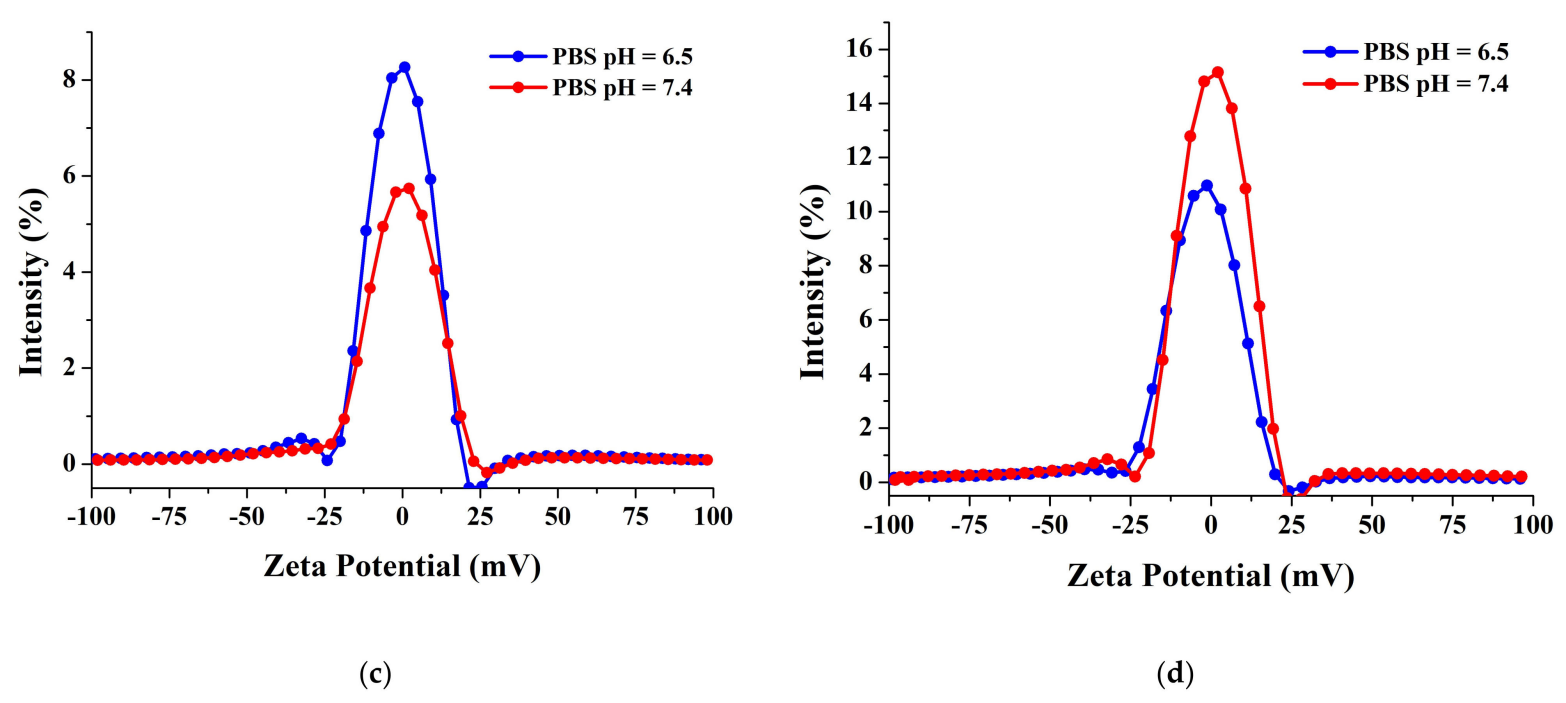
DLS analysis of drug-loaded SQ-PEG_1500_-NH-Boc micelles at 1.25 mg/mL in PBS with pH values of 6.5 and 7.4 at an ambient temperature of 25 °C. (**a**) Particle size distributions of MTx-loaded SQ-PEG_1500_-NH-Boc micelles; (**b**) particle size distributions of Cyt-loaded SQ-PEG_1500_-NH-Boc micelles; (**c**) zeta potentials of MTx-loaded SQ-PEG_1500_-NH-Boc micelles; (**d**) zeta potentials of Cyt-loaded SQ-PEG_1500_-NH-Boc micelles. Data are presented as mean ± SD (*n* = 3).

Drug-loaded SQ-PEG_1500_-NH-Boc micelles were analyzed by DLS experiments in terms of particle size and zeta potentials in PBS with pH values of 6.5 (tumoral) and 7.4 (physiological) at an ambient temperature of 25 °C (Figure 7 and Table 3). The solutions containing the drug-loaded SQ-PEG_1500_-NH-Boc micelles were analyzed at three different concentrations above the CMC value of 0.154 mg/mL (0.75, 1.00, and 1.25 mg/mL) to observe a dependence of their properties with the variation of the concentration, but for simplicity, only the results for the concentration of 1.25 mg/mL are discussed; the rest of the results can be found in the Supplementary Materials (Figures S16 and S17, Tables S2 and S3).

The data obtained from particle size measurements of drug-loaded SQ-PEG_1500_-NH-Boc micelles showed broad unimodal hydrodynamic diameter distributions for MTx-loaded nanotherapeutic at both pH values (Figure 7a). Meanwhile, the Cyt-loaded nanotherapeutic presented a narrow unimodal hydrodynamic diameter distribution at a pH value of 6.5 and broad unimodal hydrodynamic diameter distributions at a pH value of 7.4 (Figure 7b). The obtained data from three distinct measurements showed that the sizes of drug-loaded SQ-PEG_1500_-NH-Boc micelles were dependent on the pH value of the media (Table 3). In the case of the MTx-loaded nanotherapeutics, the average H_d_ value at pH 6.5 (79.3 ± 5.3 nm) was higher than at pH 7.4 (56.5 ± 5.9). On the contrary, the Cyt-loaded nanotherapeutic’s average H_d_ value at pH 6.5 (438.9 ± 7.5) was lower than at pH 7.4 (551.3 ± 5.3). By comparison, the Cyt-loaded nanotherapeutic’s average H_d_ was higher than the average H_d_ of MTx-loaded micelles, but they have better homogeneity, as proved by a lower value of the polydispersity index (PDI). This phenomenon can be attributed to weaker interactions between the PEG chain and the Cyt-cargo, resulting in stronger interactions between the polymer and the solvent.

The zeta potential values of drug-loaded nanotherapeutics (Figure 7c,d) were −0.43 ± 0.1 mV (PBS 6.5) and +0.47 ± 0.2 mV (PBS 7.4) for MTx-loaded micelles, and −2.21 ± 0.1 mV (PBS 6.5) and +0.70 ± 0.4 mV (PBS 7.4) for the Cyt-loaded micelles (Table 3). The zeta potential values between −10 and + 10 mV are considered neutral [67], and the values obtained in this interval may be explained by the fact that the PEG is present on the surface of all investigated micelles as a result of the observed neutral charge.

**Figure 8:**
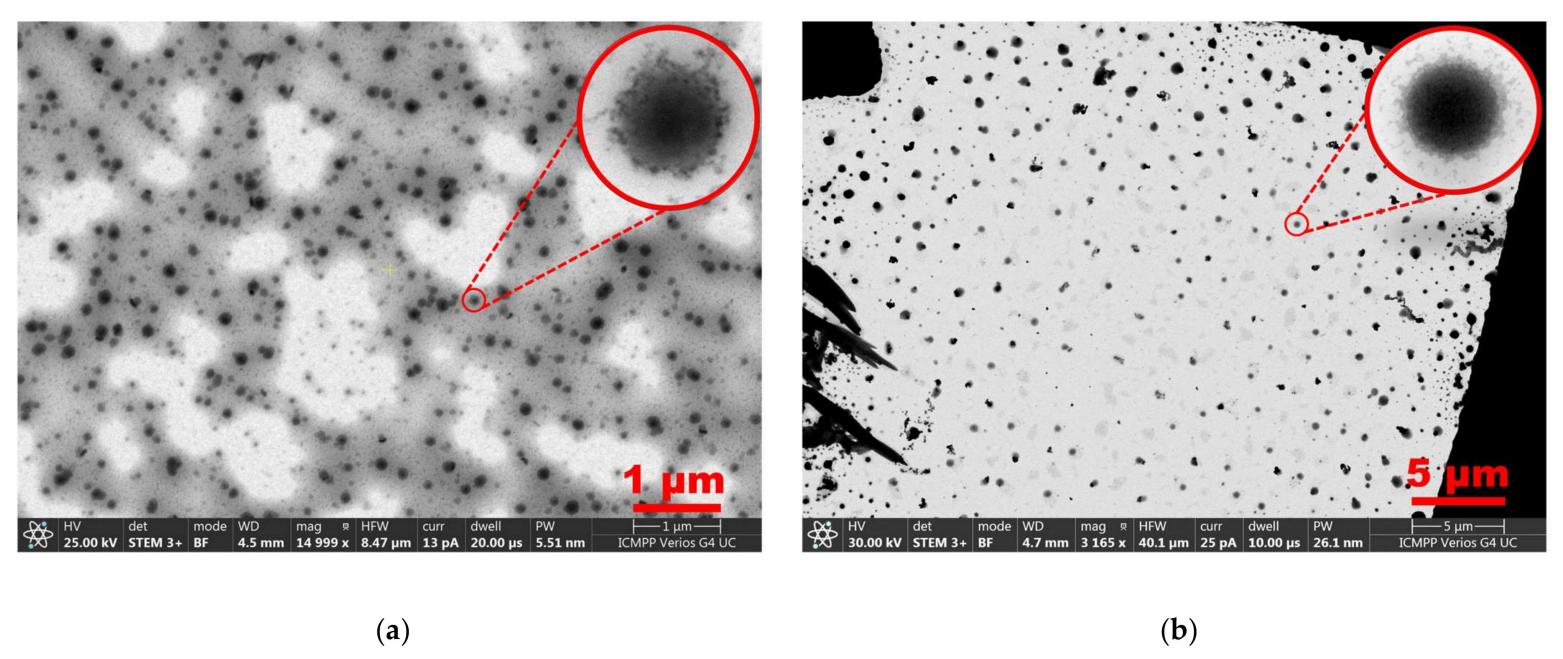

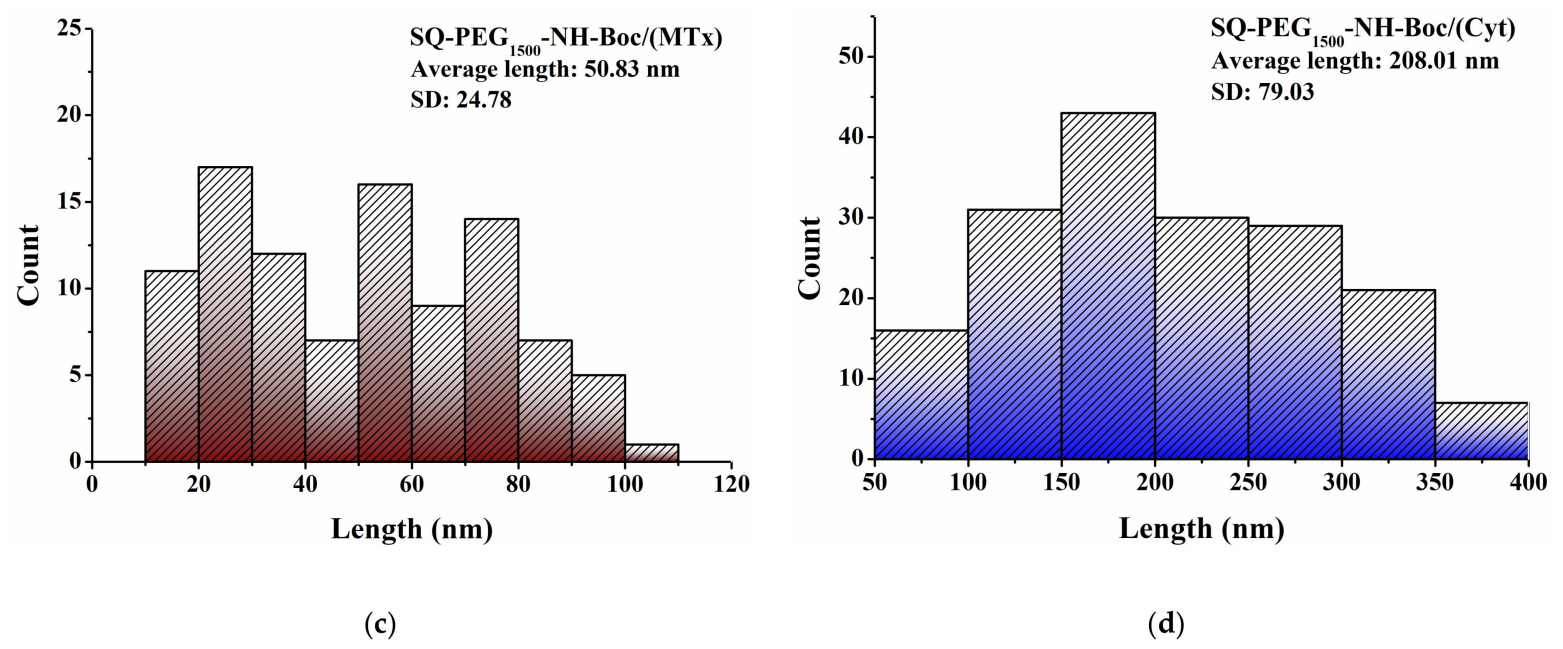
STEM morphological analysis of drug-loaded SQ-PEG_1500_-NH-Boc micelles. (**a**) STEM image of MTx-loaded micelles; (**b**) STEM image of Cyt-loaded micelles and their size distribution plots; (**c**) size distribution of MTx-loaded micelles (*n* = 120 micelles); (**d**) size distribution of Cyt-loaded micelles (*n* = 120 micelles).

The static morphologies of drug-loaded SQ-PEG_1500_-NH-Boc micelles were evaluated by STEM analysis and their size distributions (Figure 8). The STEM images of both analysed samples showed that the drug-loaded micelles have core–shell-type spherical morphologies with nanometric dimensions (Figure 8a,b). Interestingly, at a closer examination (red circles), it could be observed that MTx-loaded nanotherapeutics (Figure 8a) showed some worm-like formations on the surface of the shell and contained dense entities, which can be identified as PEG chains trapping MTx-drug molecules. This observation was confirmed by the neutral charge density observed in zeta potential values from DLS measurements. Also, the Cyt-loaded nanotherapeutics (Figure 8b) showed the same worm-like formations at the surface of micelles, but, in this case, their densities were not as dense as in the previous case, meaning that the Cyt molecules were trapped inside the micellar architectures, as proved by a homogeneous dense core. The size distributions of drug-loaded micelles were determined by measuring the sizes of the nano-entities in the STEM images using ImageJ software v1.54d [47] and were graphically plotted, as shown in Figure 8c,d. The MTx-loaded micelles presented an average size of 50.83 ± 24.78 nm with a size distribution between 10 and 110 nm (Figure 8c). Meanwhile, Cyt-loaded micelles’ average size was 208.01 ± 79.03 nm with a size distribution between 50 and 400 nm (Figure 8d). The results obtained from the STEM analysis agree with the results obtained from the DLS analysis, confirming the big difference between the dimensions of both studied systems.

As proven by the STEM analysis, both nanotherapeutics have core–shell-type spherical morphologies with homogeneous size distribution and nanometric dimensions, which make them suitable for biomedical applications.

### 3.6. In Vitro Drug Release from SQ-PEG_1500_-NH-Boc Micelles

**Figure 9:**
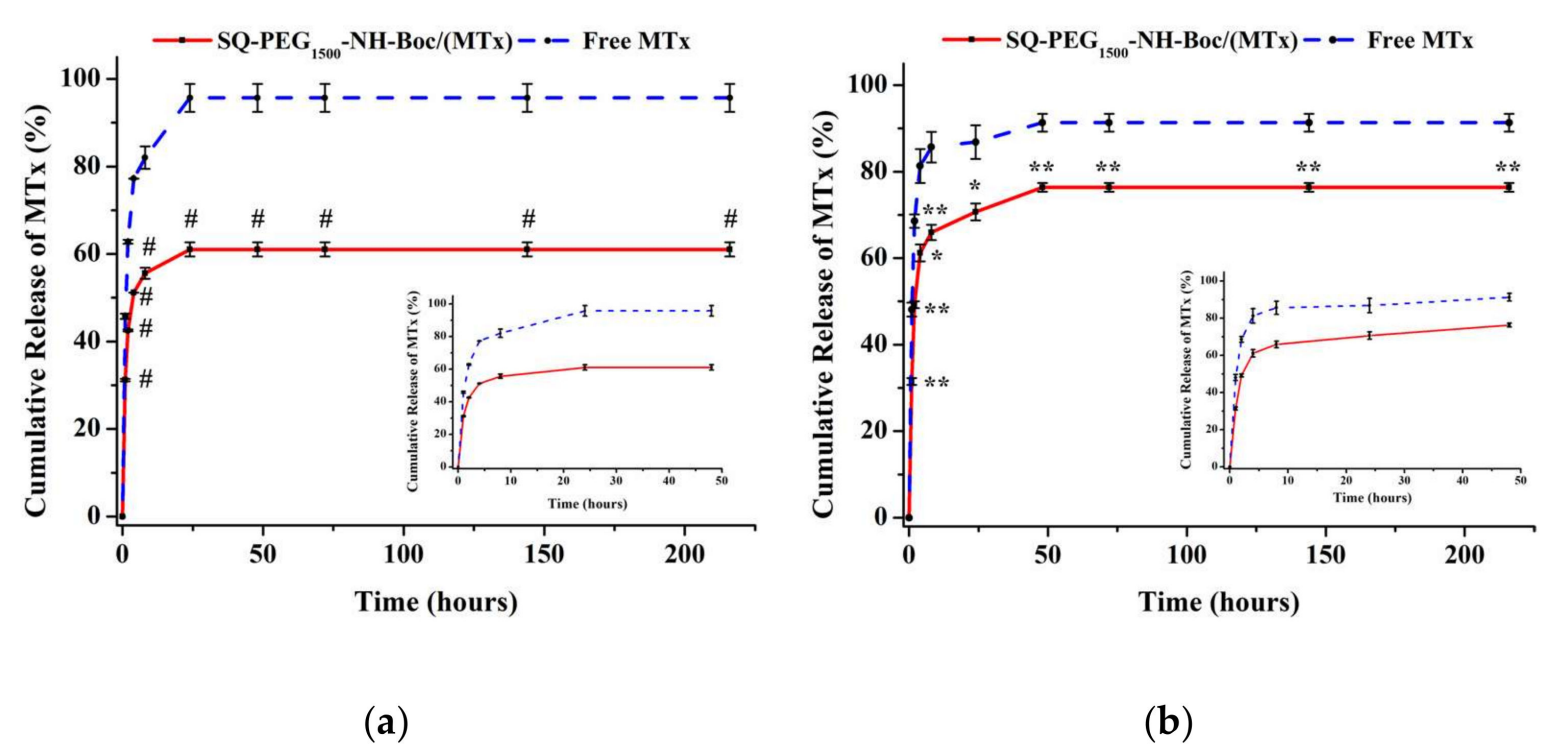
In vitro cumulative release of MTx from the free MTx solution and MTx-encapsulated SQ-PEG_1500_-NH-Boc micelles. (**a**) In simulated physiological conditions (PBS with pH of 7.4 and 37 °C); (**b**) in simulated tumoral conditions (PBS with pH 6.5 and 39 °C). The experiment was carried out for 9 days, and the results are expressed as means ± SEM (*n* = 3). * *p* < 0.05, ** *p* < 0.01, and # *p* > 0.05 (ns) by Student’s *t*-test.

**Figure 10:**
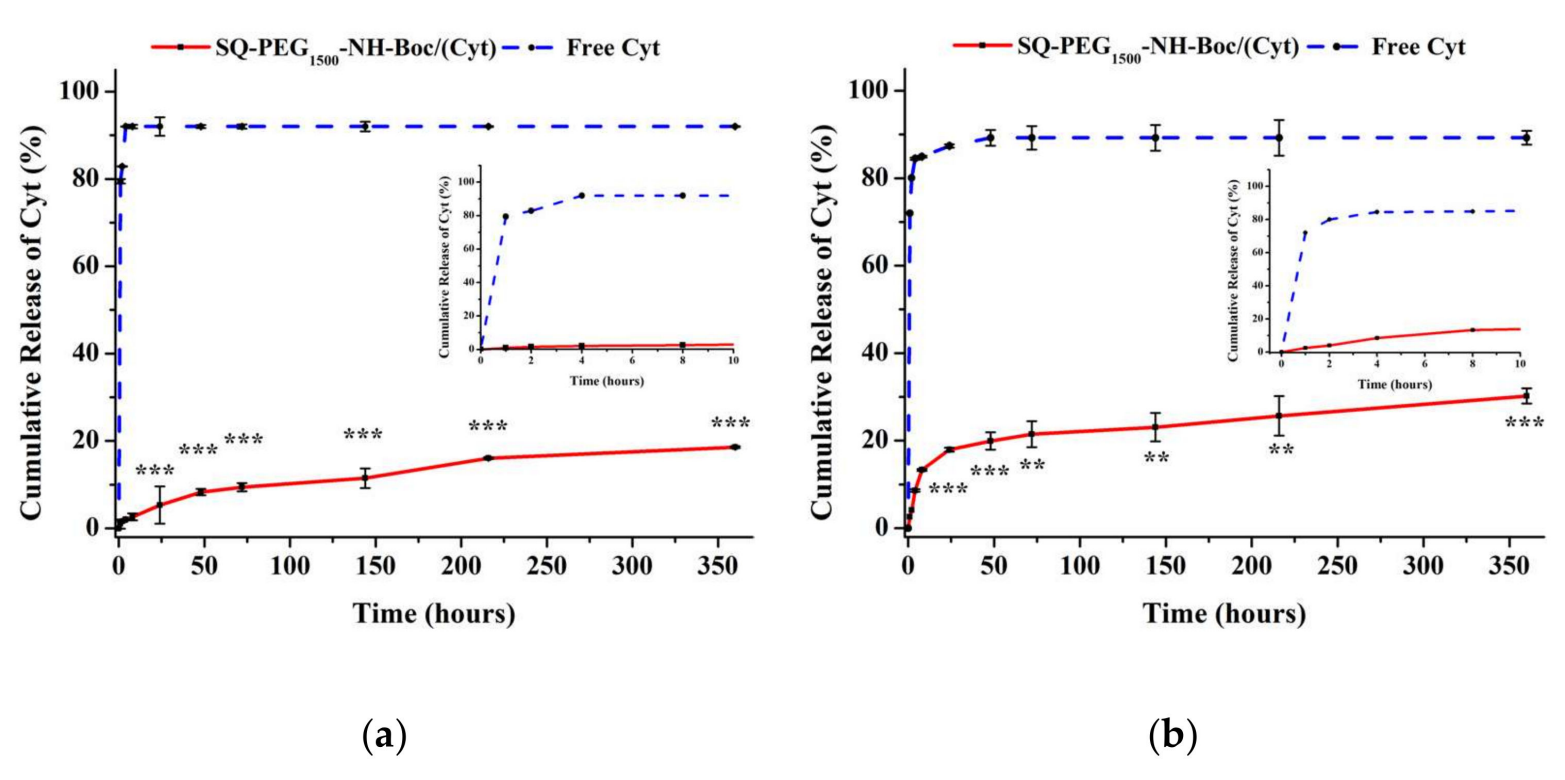
In vitro cumulative release of Cyt from the free Cyt solution and Cyt-encapsulated SQ-PEG_1500_-NH-Boc micelles. (**a**) In simulated physiological conditions (PBS with pH of 7.4 and 37 °C); (**b**) in simulated tumoral conditions (PBS with pH 6.5 and 39 °C). The experiment was carried out for 15 days, and the results are expressed as means ± SEM *(n* = 3*).* ** *p* < 0.01, *** *p* < 0.001, by Student’s *t*-test.

The in vitro drug release behaviour of MTx/Cyt-loaded SQ-PEG_1500_-NH-Boc micelles was evaluated in 1× PBS (pH 7.4 and 6.5) at 37 °C and 39 °C, respectively, to simulate both physiological and tumour tissue conditions. Control experiments using free drugs (MTx and Cyt) were also carried out in similar conditions, and the results are shown in Figure 9 for MTx and Figure 10 for Cyt. In the case of the MTx study, at pH 7.4 and 37 °C (Figure 9a), for free MTx, complete diffusion across the dialysis membrane was found to occur within 24 h. Interestingly, the MTx-loaded nanotherapeutic release behaviour reached a maximum of 60% in the same amount of time, but after 216 h, the cumulative release of MTx remained unchanged. This phenomenon can be explained because in physiological conditions, the SQ-PEG_1500_-NH-Boc micelles can protect the cargo by limiting the release of the drug to 60% and keeping the rest of it for the targeted site. In the case of the MTx study, by mimicking the tumoral conditions (pH 6.5 and 39 °C) (Figure 9b), the release behaviour of the free MTx was completely changed by a two-stage kinetic mechanism involving a burst release of approximately 80% of the drug within 4 h and a slow-release profile until 90% within 48 h. The release behaviour of MTx from SQ-PEG_1500_-NH-Boc micelles has a similar two-stage kinetic mechanism as the free MTx, but in this case, in the burst release stage, the cumulative release of MTx was around 60%; meanwhile, in the slow-release stage, 75% of the cumulative release of MTx was within 48 h.

The Cyt-release study revealed that due to its hydrophilicity, this drug behaves differently than the MTx. At physiological conditions (Figure 10a), for the free Cyt, a complete diffusion against the dialysis membrane was observed within 4 h, with the burst-release type kinetic reaching approximately 90% of cumulative drug release. Contrarily, the Cyt-loaded nanotherapeutic showed a very slow sustained release, which after 360 h reached approximately 20% of cumulative drug release. Also, in this case, the protective property of the SQ-PEG_1500_-NH-Boc micelles for the carried drug could be observed.

In tumoral conditions (Figure 10b), the release behaviour is similar to physiological conditions with the exception of the Cyt-loaded nanotherapeutic cumulative release, which reached approximately 30% after 360 h. The weak capacity of SQ-PEG_1500_-NH-Boc micelles to release the encapsulated Cyt may be attributed mainly to the hydrophilicity of the drug and secondly to the H-bonding interactions between OH groups from Cyt and oxygen atoms from the repetitive unit from PEG. These observations are also noted by other researchers in the scientific literature [71].

### 3.7. Drug Release Kinetics Using Different Mathematical Models

To gain a deeper understanding of the release process of MTx and Cyt from the SQ-PEG_1500_-NH-Boc micelles in different pH mediums and to assess the key factors governing this process, the kinetic data were fitted to various mathematical models, including the zero-order, first-order, Higuchi, Hixson–Crowell, and Korsmeyer–Peppas models. This analysis was conducted during the first stage (1–8 h) of the release curves (Figure S18) for both MTx and Cyt drugs.

By analysing the correlation coefficients obtained from the mathematical models applied, different behaviours were observed among the studied samples (Table 4). The mathematical models with the highest correlation coefficients (>0.8) were selected to identify the most appropriate release model for this type of system. Thus, for the MTx drug, the suitable models are the Higuchi and Korsmeyer–Peppas models, where the correlation coefficients obtained for the Higuchi model ranged from 0.83 to 0.89, while the best-fit model was the Korsmeyer–Peppas model, with correlation coefficients ranging from 0.89 to 0.93. To examine the release mechanism, the experimental release data for MTx-loaded SQ-PEG_1500_-NH-Boc micelles were plotted as the logarithm of cumulative % drug release versus the logarithm of time to determine the release exponent (***n***). All the samples under study displayed ***n*** values < 0.5, indicating a Fickian diffusion drug release mechanism [72].

The applied mathematical models are suitable for the Cyt-loaded micelles, with correlation coefficients ranging from 0.81 to 0.97. The best-fitted models are Higuchi and Korsmeyer–Peppas, where the release exponent (*n*) values obtained for the Cyt drug release provide valuable insights into the release mechanism. The release study of Cyt-loaded micelles in physiological conditions revealed a Fickian diffusion drug release mechanism (*n* = 0.45), while the release study of Cyt-loaded micelles in tumoral conditions exhibited a non-Fickian diffusion mechanism (*n* = 0.63) [73]. Furthermore, the release constant (k) is directly proportional to the diffusion constant, and its value indicates the rate of drug release (higher values indicate a faster release rate, while lower values suggest a slower release rate). In our study, the lowest release rates were observed for Cyt-loaded micelles (0.01 and 0.03), and the highest were observed for the MTx-loaded micelles (0.35 and 0.38) [73]. These variations in release rates can be ascribed to the hydrophilic nature of the encapsulated drug, as well as the distinct interactions between the drug and the SQ-PEG_1500_-NH-Boc micelles, as reported elsewhere [74].

### 3.8. In Vitro Antitumor Activity of the Studied Nanotherapeutics

**Figure 11:**
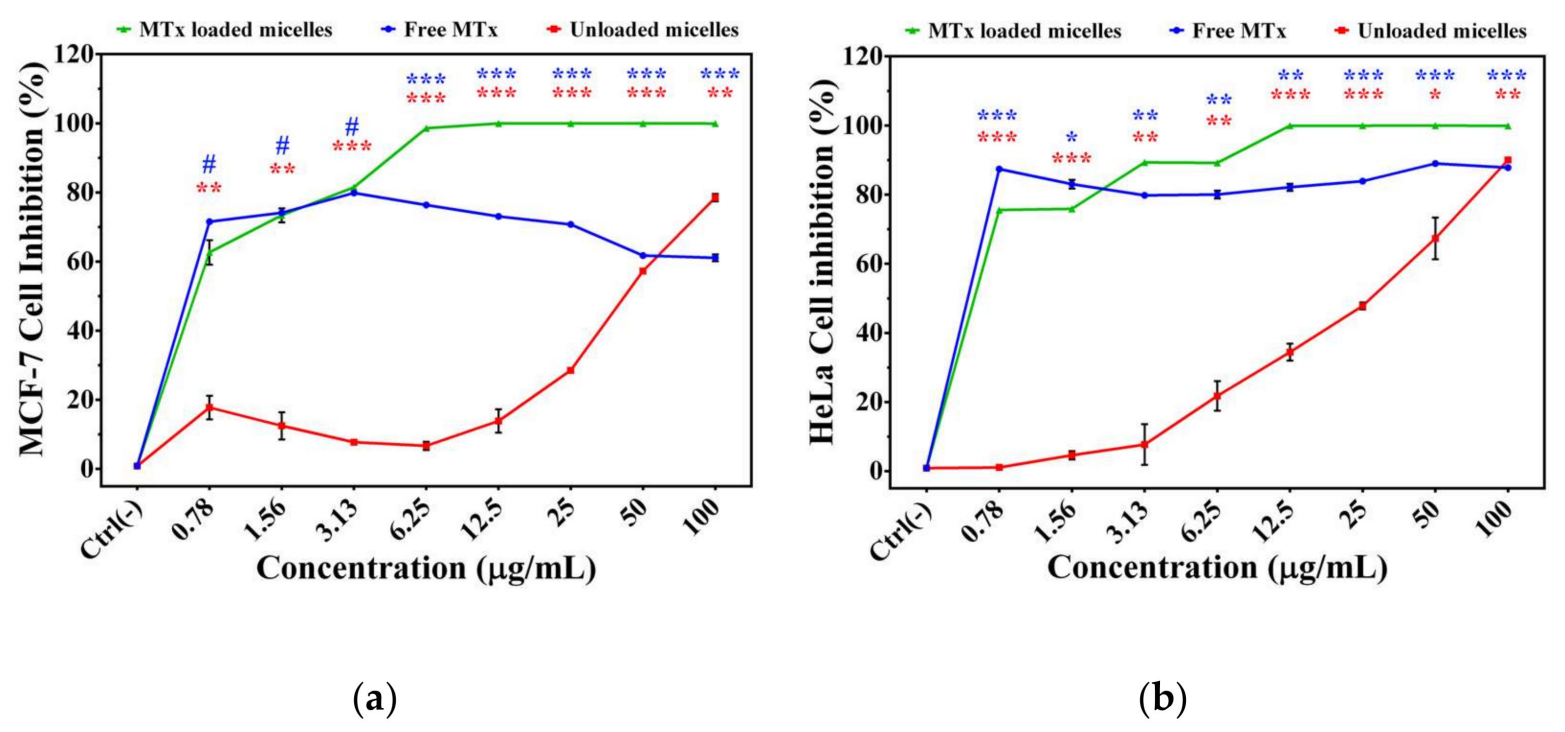
In vitro antitumor activity of free MTx and MTx-loaded SQ-PEG_1500_-NH-Boc micelles and unloaded SQ-PEG_1500_-NH-Boc micelles at various concentrations of MTx against (**a**) MCF-7 cells and (**b**) HeLa cells after incubation for 72 h. Results are reported as % of cell inhibition based on the untreated control cells normalized to 0% and are expressed as means ± SD (*n* = 3). * *p* < 0.05, ** *p* < 0.01, *** *p* < 0.001, and # *p* > 0.05 (ns) (for MTx-loaded micelles vs. free MTx) and * *p* < 0.05, ** *p* < 0.01, *** *p* < 0.001 (for MTx-loaded micelles vs. unloaded micelles) by Student’s *t*-test.

**Figure 12:**
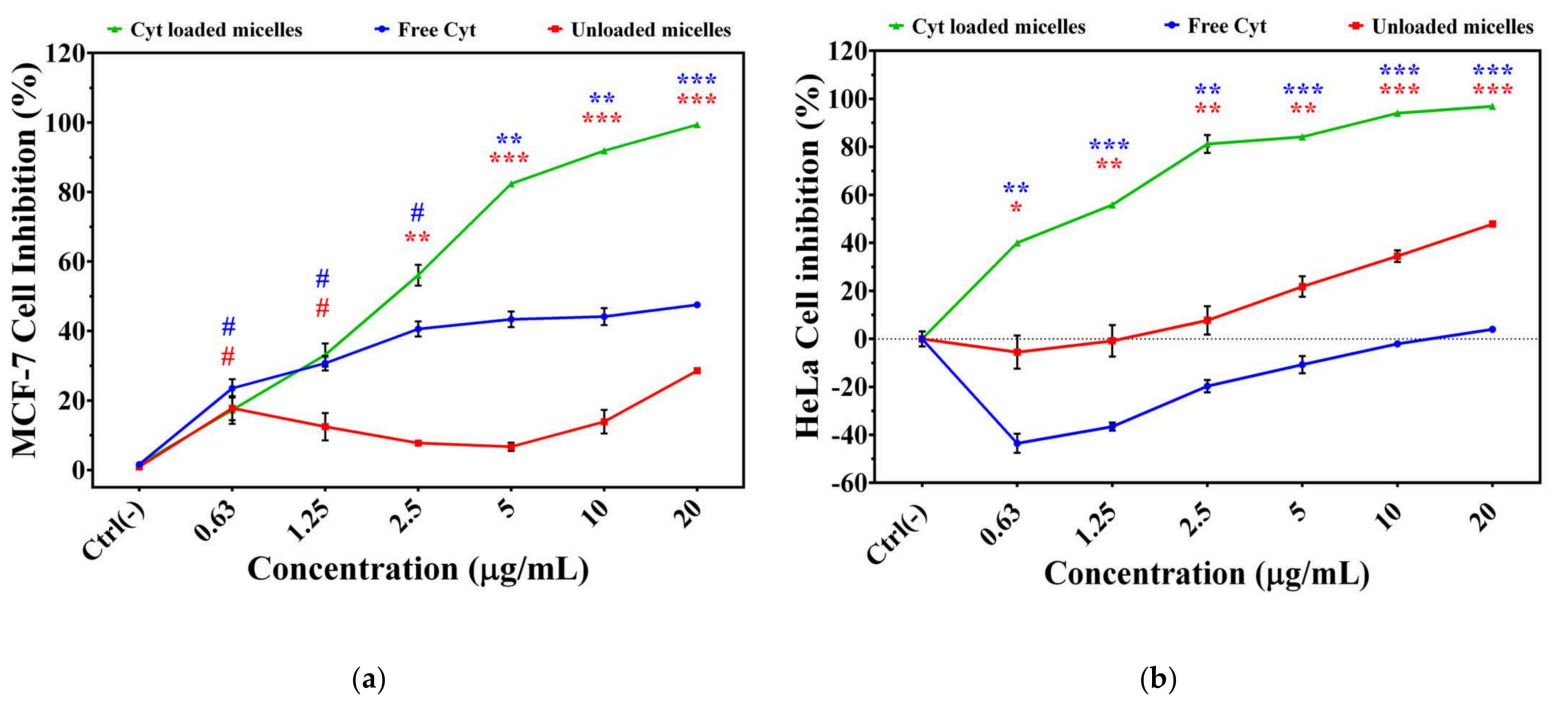
In vitro antitumor activity of free Cyt, Cyt-loaded SQ-PEG_1500_-NH-Boc micelles, and unloaded SQ-PEG_1500_-NH-Boc micelles at various concentrations of Cyt against (**a**) MCF-7 cells and (**b**) HeLa cells after incubation for 72 h. Results are reported as % of cell inhibition based on the untreated control cells normalized to 0% and are expressed as means ± SD (*n* = 3). ** *p* < 0.01, *** *p* < 0.001, and # *p* > 0.05 (ns) (for Cyt-loaded micelles vs. free Cyt) and * *p* < 0.05, ** *p* < 0.01, *** *p* < 0.001, and # *p* > 0.05 (ns) (for Cyt-loaded micelles vs. unloaded micelles) by Student’s *t*-test.

To investigate the antitumor efficiency of the MTx/Cyt-encapsulated SQ-PEG_1500_-NH-Boc micelles, the viability of two different cancer cell lines, including MCF-7 cells and HeLa cells, was measured using the CellTiter-Glo assay. The concentration of MTx ranged from 0 to 100 µg/mL (Figure 11); meanwhile, the concentration of Cyt was between 0.63 and 20 µg/mL (Figure 12). In both experiments, the concentration of unloaded SQ-PEG_1500_-NH-Boc micelles was calculated as the concentration in the drug-loaded SQ-PEG_1500_-NH-Boc micelles based on the weight ratio between the encapsulated drug and SQ-PEG_1500_-NH-Boc micelles (more information can be found in the Supplementary Materials in Table S4). As shown in Figure 11, as expected, the amphiphilic SQ-PEG_1500_-NH-Boc assembly showed significant activity against the MCF-7 and HeLa cell lines at higher concentrations, whereas, at the concentration of 100 µg/mL (3.9 mg/mL as SQ-PEG_1500_-NH-Boc concentration), the inhibition was 80% for the MCF-7 cells and 90% for the HeLa cells. This antitumor activity of SQ-PEG_1500_-NH-Boc at higher concentrations is due to the squalene moiety that is known to possess anticancer properties, as this was already discussed elsewhere [75,76]. On the contrary, the free MTx and MTx-loaded micelles inhibited the growth of MCF-7 and HeLa cells in a similar manner at low concentrations (below 3.13 µg/mL) in both experiments. The difference between the antitumor activity of free MTx and MTx-loaded micelles starts to be observed after the concentration of 6.25 µg/mL for the MCF-7 cell line (Figure 11a) and 12.5 µg/mL for the HeLa cell line (Figure 11b), where the MTx-loaded nanotherapeutic showed an increase in the tumour cell inhibition, and this improvement being more visible in the case of MCF-7 cell experiment (Figure 11a), where the cell inhibition of free MTx starts to decrease to a value of 60% at 100 µg/mL. Meanwhile, the cell inhibition of the MTx-loaded nanotherapeutic reached the maximum cell inhibition at the same concentration. Regarding the HeLa cell inhibition (Figure 11b), the MTx-loaded nanotherapeutic also reached the maximum inhibition at 100 µg/mL and, interestingly, the free MTx showed a higher inhibition value (90%) in comparison with the MCF-7 cell line at the same concentration, and this observation demonstrated the affinity of the free MTx for the HeLa cell line instead of MCF-7. Although the MTx-loaded nanotherapeutic did not have this property, it is efficient on both cell lines.

The activity of the SQ-PEG_1500_-NH-Boc assembly against MCF-7 cells and HeLa cells during the experiment involving the second studied drug (Cyt) is shown in Figure 12. The unloaded micelles demonstrated a similar behaviour as in the previous experiment on the studied concentrations between 0.63 µg/mL and 20 µg/mL (expressed as the concentration of Cyt) or between 10.69 µg/mL and 339.27 µg/mL (expressed as the concentration of SQ-PEG_1500_-NH-Boc; more information can be found in the Supplementary Materials in Table S5). In this case, the highest activity of the SQ-PEG_1500_-NH-Boc assembly was observed against the HeLa cells at the highest tested concentration, where the inhibition was approximately 40%. Regarding the antitumor activity of free Cyt, it is obvious that this drug has an affinity for the MCF-7 cells, where the maximum tested concentration of 20 µg/mL presented an inhibition value of approximately 40% (Figure 12a). Meanwhile, for the HeLa cells, a proliferation until the concentration of 10 µg/mL was observed, and the maximum inhibition value was approximately 10% at the highest concentration (Figure 12b). The proliferation observed in the HeLa cells during the inhibition assay, especially in the case of the commercially available Cyt, can be attributed to the CellTiter-Glo reagent. This reagent estimates cell viability based on a direct relationship between the number of cells and the amount of adenosine triphosphate (ATP). However, in certain situations, cellular metabolism and ATP synthesis may fluctuate in response to treatment. Consequently, treatment with Cyt can result in an increase in mitochondrial membrane potential and ATP levels [77]. Moreover, treatment may lead to a gradual development of resistance to Cyt, as low inhibition may not be apparent at 48 h but becomes evident after 3 days. Cyt-resistant cells exhibit increased mitochondrial mass and ATP production [78]. This could explain the elevated ATP levels and the subsequent proliferation effect in Figure 12b.

Remarkably, the Cyt-loaded nanotherapeutic showed improved activity against both tumoral cell lines, possessing an affinity for the HeLa cells, which showed an inhibition value of 80% at a concentration of 2.5 µg/mL (Figure 12b), and the same inhibition value was obtained at 5 µg/mL in the case of MCF-7 cells (Figure 12a). Interestingly, it can be observed that by encapsulating the antitumor drugs, MTx and Cyt, into SQ-PEG_1500_-NH-Boc micelles, their biological properties improved and, in some cases, their affinities for specific cancerous cells altered. These observations strengthen the importance of using SQ-PEG_1500_-NH-Boc micelles as drug delivery systems for commercial drugs with antitumor applications.

## 4. Conclusions

In the presented study, amphiphilic PEGylated squalene sequences were utilized to prepare micellar architectures encapsulating commercially available antitumoral drugs, MTx and Cyt, through ultrasound-assisted dropping and solvent evaporation methods. Encapsulation efficiencies were satisfactory, with percentages of 54.02 ± 0.3033 for MTx and 44.60 ± 6.86 for Cyt. The drug-encapsulated micelles exhibited spherical “core-shell” entities with nanometric dimensions and well-distributed sizes (50.83 ± 24.78 nm for MTx-loaded micelles and 208.01 ± 79.03 nm for Cyt-loaded micelles). Furthermore, the studied nanotherapeutics demonstrated favourable stability and sustained in vitro release for 48 h in the case of MTx (78% in tumoral conditions and 60% in physiological conditions) and 360 h for Cyt (30% in tumoral conditions and 20% in physiological conditions). Interestingly, the in vitro drug release from PEGylated squalene micelles showed dependence on the biological environment (physiological or tumoral), indicating that these micelles can shield the cargo during bloodstream circulation until reaching the target delivery site (tumoral site). Furthermore, the applied release kinetics, as analysed with mathematical models, demonstrated that the most suitable model for this type of system was the Korsmeyer–Peppas model. The results obtained from this study confirm that the mechanism of drug release depends on both the nature of the drug (hydrophilic or hydrophobic) and the environmental medium (physiological or tumoral).

In vitro, cytotoxicity studies on MCF-7 and HeLa cell lines revealed that the MTx- and Cyt-loaded nanotherapeutics exhibited promising high tumour inhibition. At concentrations of 100 µg/mL (the MTx concentration), MTx-loaded micelles achieved 100% inhibition efficiency on both cell lines, while Cyt-loaded micelles, at concentrations of 20 µg/mL (the Cyt concentration), also achieved 100% inhibition on both cell lines. These encouraging results suggest the potential use of MTx- and Cyt-loaded PEGylated squalene micelles as effective drug-delivery systems for further in vivo testing and potential consideration for antitumoral therapy in clinical trials.

## Figures and Tables

**Table 1:** Colloidal characteristics of SQ-PEG_1500_-NH-Boc micelles in PBS solutions (1.25 mg/mL, 23 °C). Data are presented as mean ± SD (*n* = 3).

PBS pH	CMC (mg/mL)	Average Hd (nm)	PDI	Average Zeta Potential (mV)
7.4	0.154	36.4 ± 2.6	0.252 ± 0.017	−2.92 ± 0.67
6.5		44.6 ± 4.4	0.440 ± 0.026	−6.54 ± 0.17

**Table 2:** Encapsulation efficiency (%EE) and drug loading capacity (%DL) of MTx and Cyt in SQ-PEG_1500_-NH-Boc micelles.

Sample	^1^ (%EE) ± SD	^1^ (%DL) ± SD
SQ-PEG_1500_-NH-Boc/(MTx)	54.02 ± 0.303	3.99 ± 0.086
SQ-PEG_1500_-NH-Boc/(Cyt)	44.60 ± 6.862	3.48 ± 1.715

^1^ The results are presented as mean values of triplicates ± SD.

**Table 3:** Colloidal characteristics of drug-loaded SQ-PEG_1500_-NH-Boc micelles at 1.25 mg/mL in PBS with pH values of 6.5 and 7.4 at an ambient temperature of 25 °C.

Sample	pH	^1^ Average H_d_ (nm) ± SD	^1^Average PDI ± SD	^1^ Average Zeta Potential (mV) ± SD
SQ-PEG_1500_-NH-Boc/(MTx)	6.5	79.30 ± 5.30	0.30 ± 0.03	−0.43 ± 0.10
	7.4	56.50 ± 5.90	0.46 ± 0.01	+0.47 ± 0.22
SQ-PEG_1500_-NH-Boc/(Cyt)	6.5	438.90 ± 7.50	0.25 ± 0.02	−2.21 ± 0.11
	7.4	551.30 ± 5.30	0.26 ± 0.05	+0.70 ± 0.35

^1^ Data are presented as mean ± SD (*n* = 3).

**Table 4:** The data obtained by applying various mathematical models to analyse the release kinetics of MTx and Cyt during the initial phase (1–8 h) of the release process.

Sample	pH	Zero-Order		First-Order		Higuchi		Hixson–Crowell		Korsmeyer–Peppas		
		R^2^	K_0_	R^2^	K_t_	R^2^	K_H_	R^2^	k	R^2^	k	*n*
Loaded MTx	6.5	0.74	4.27	0.67	−0.09	0.85	17.84	0.79	−0.11	0.89	0.35	0.35
	7.4	0.79	3.09	0.73	−0.07	0.89	12.79	0.82	−0.07	0.93	0.38	0.28
Loaded Cyt	6.5	0.85	0.95	0.81	−0.17	0.91	3.84	0.85	−0.015	0.93	0.03	0.63
	7.4	0.91	0.21	0.82	−0.12	0.97	0.85	0.91	−0.003	0.97	0.01	0.45

Where **R^2^** is the correlation coefficient, **K** is the proportionality constant, and ***n*** is the release exponent.

## Data Availability

Data are available upon request.

## Supplementary Materials

The following supporting information can be downloaded at https://www.mdpi.com/article/10.3390/polym15214225/s1, Figure S1: ^1^H-NMR spectrum of SQ-COOH; Figure S2: ^13^C-NMR spectrum of SQ-COOH; Figure S3: ATR-FTIR spectrum of SQ-COOH; Figure S4: ESI-MS spectrum of SQ-COOH; Figure S5: ^1^H-NMR spectrum of H_2_N-PEG_1500_-NH-Boc; Figure S6: ^13^C-NMR spectrum of H_2_N-PEG_1500_-NH-Boc; Figure S7: ATR-FTIR spectrum of H_2_N-PEG_1500_-NH-Boc; Figure S8: MALDI-TOF/TOF MS spectrum of H_2_N-PEG_1500_-NH_2_ and H_2_N-PEG_1500_-NH-Boc; Figure S9: ^1^H-NMR spectrum of SQ-PEG_1500_-NH-Boc; Figure S10: ^13^C-NMR spectrum of SQ-PEG_1500_-NH-Boc; Figure S11: ATR-FTIR spectrum of SQ-PEG_1500_-NH-Boc; Figure S12: MALDI-TOF/TOF MS spectrum of SQ-PEG_1500_-NH-Boc; Figure S13: DLS analysis of SQ-PEG_1500_-NH-Boc micelles in PBS solutions (pH 6.5 and pH 7.4) at three concentrations; Table S1: Colloidal characteristics of SQ-PEG_1500_-NH-Boc micelles in PBS solutions (pH 6.5 and pH 7.4) at 23 °C at three concentrations; Figure S14: Calibration curves of MTx in PBS (pH 6.5 and pH 7.4) by UV-Vis; Figure S15: Calibration curves of Cyt in PBS (pH 6.5 and pH 7.4) by UV-Vis; Figure S16: DLS analysis of MTx-loaded SQ-PEG_1500_-NH-Boc micelles in PBS solutions (pH 6.5 and 7.4) at three concentrations; Table S2: Colloidal characteristics of MTx-loaded SQ-PEG_1500_-NH-Boc micelles in PBS solutions (pH 6.5 and 7.4) at 23 °C in three concentrations; Figure S17: DLS analysis of Cyt-loaded SQ-PEG_1500_-NH-Boc micelles in PBS solutions (pH 6.5 and 7.4) at three concentrations; Table S3: Colloidal characteristics of Cyt-loaded SQ-PEG_1500_-NH-Boc micelles in PBS solutions (pH 6.5 and 7.4) at 23 °C in three concentrations; Figure S18: Linear fitting of the mathematical models applied for the release of MTx and Cyt from SQ-PEG_1500_-NH-Boc micelles; Table S4: Concentrations of the MTx-loaded SQ-PEG_1500_-NH-Boc micelles, which were used for the in vitro antitumor activity experiments calculated as MTx and SQ-PEG_1500_-NH-Boc; Table S5: Concentrations of the Cyt-loaded SQ-PEG_1500_-NH-Boc micelles, which were used for the in vitro antitumor activity experiments calculated as Cyt and SQ-PEG_1500_-NH-Boc.
