# Supplementary material for: Novel Nanotherapeutic Systems Based on PEGylated Squalene Micelles for Enhanced In Vitro Activity of Methotrexate and Cytarabine
Source: Polymers (Basel). 2023 Oct 25;15(21):4225. doi: 10.3390/polym15214225 (PMC10650902; doi:10.3390/polym15214225)
Supplement: Supplementary file 1 [file polymers-15-04225-s001.zip › polymers-2674273-supplementary.pdf]

polymers

Article

Supplementary Material for: Novel Nanotherapeutic Systems Based on PEGylated Squalene Micelles for Enhanced In Vitro Activity of Methotrexate and Cytarabine

Bogdan-Florin Craciun *, Isabela-Andreea Sandu, Dragos Peptanariu and Mariana Pinteala *

Centre of Advanced Research in Bionanoconjugates and Biopolymers, "Petru Poni" Institute of Macromolecular Chemistry, 41A Grigore Ghica Voda Alley, 700487 Iasi, Romania; sandu.isabela@icmpp.ro (I.-A.S.); dragos.peptanariu@icmpp.ro (D.P.)
* Correspondence: craciun.bogdan@icmpp.ro (B.-F.C.); pinteala@icmpp.ro (M.P.); Tel.: +40-332-880-050-400 (B.-F.C.); +40-332-880-050-554 (M.P.)

Table of contents:

**1. Structural characterization of SQ-COOH**	
1.1. ^{1}H-NMR spectrum	Figure S1
1.2. ^{13}C-NMR spectrum	Figure S2
1.3. ATR-FTIR spectrum	Figure S3
1.4. ESI-MS spectrum	Figure S4
**2. Structural characterization of H_2N-PEG_{1500}-NH-Boc**	
2.1. ^{1}H-NMR spectrum	Figure S5
2.2. ^{13}C-NMR spectrum	Figure S6
2.3. ATR-FTIR spectrum	Figure S7
2.4. ESI-MS spectrum	Figure S8
**3. Structural characterization of SQ-PEG_{1500}-NH-Boc**	
3.1. ^{1}H-NMR spectrum	Figure S9
3.2. ^{13}C-NMR spectrum	Figure S10
3.3. ATR-FTIR spectrum	Figure S11
3.4. MALDI-TOF/TOF MS spectrum	Figure S12
**4. Methods**	
4.1. DLS analysis of SQ-PEG_{1500}-NH-Boc in PBS solutions	Figure S13<br>Table S1
4.2. Calibration curves of MTx and Cyt in PBS solutions	
4.2.1. Calibration curves of MTx	Figure S14
4.2.2. Calibration curves of Cyt	Figure S15
4.3. DLS analysis of drug-loaded SQ-PEG_{1500}-NH-Boc micelles in PBS solutions	
4.3.1. DLS analysis of MTx-loaded micelles	Figure S16<br>Table S2
4.3.2. DLS analysis of Cyt-loaded micelles	Figure S17<br>Table S3
4.4. The study of MTx and Cyt release kinetics on mathematical models	Figure S18
4.5. Samples concentration for the *in-vitro* antitumor experiments	
4.5.1. MTx-loaded SQ-PEG_{1500}-NH-Boc micelles	Table S4
4.5.2. Cyt-loaded SQ-PEG_{1500}-NH-Boc micelles	Table S5

1. Structural characterization of SQ-COOH

1.1. ^{1}H-NMR spectrum

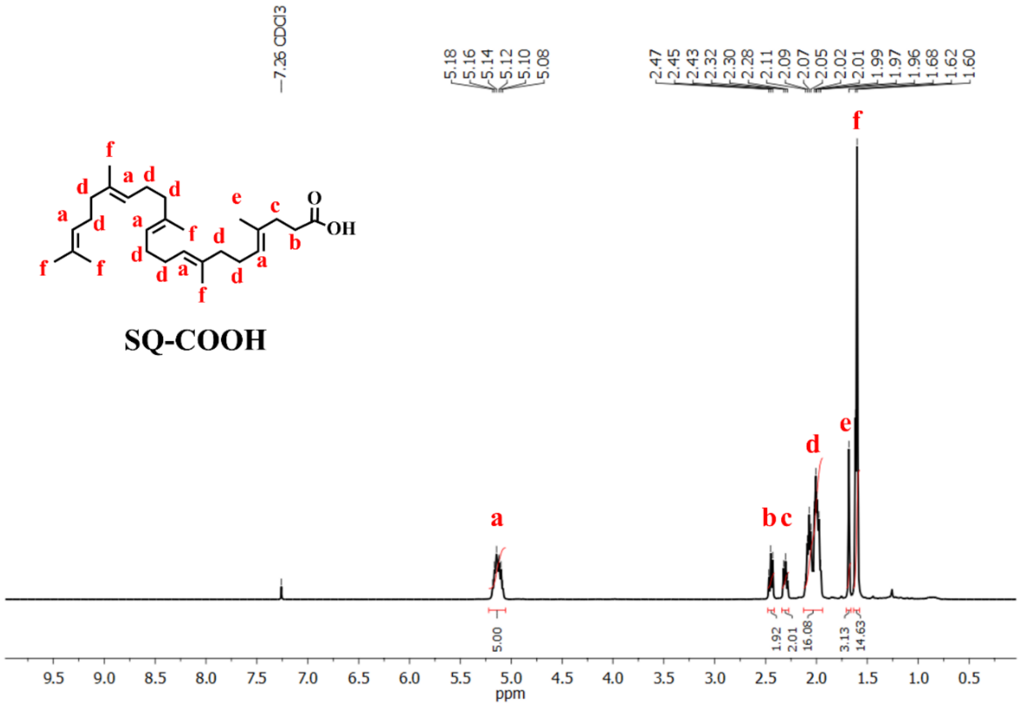

Figure S1. ^{1}H-NMR ($CDCl_3$, 400 MHz) spectrum of SQ-COOH.

1.2. ^{13}C-NMR spectrum

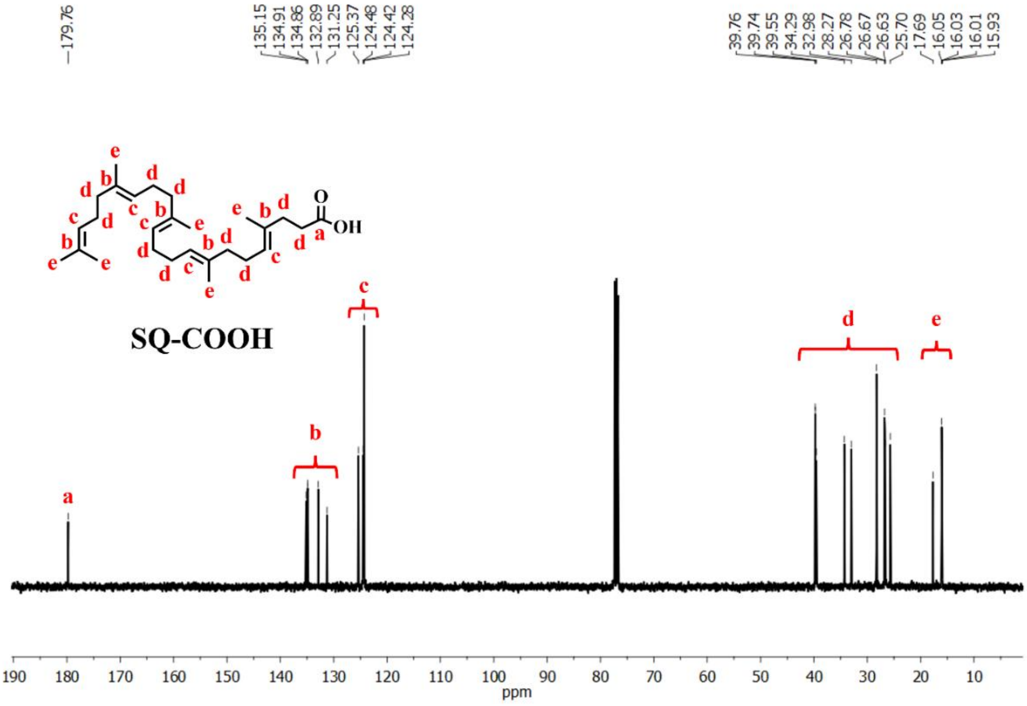

Figure S2. ^{13}C-NMR ($CDCl_3$, 400 MHz) spectrum of SQ-COOH.

1.3. ATR-FTIR spectrum

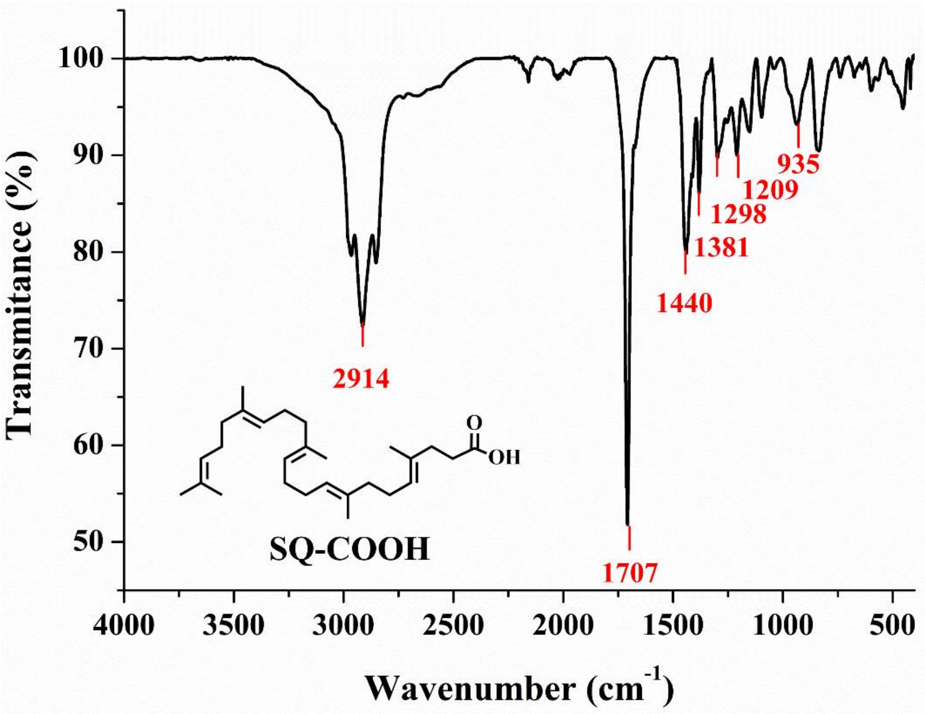

Figure S3. ATR-FTIR spectrum of SQ-COOH.

1.4. ESI-MS spectrum

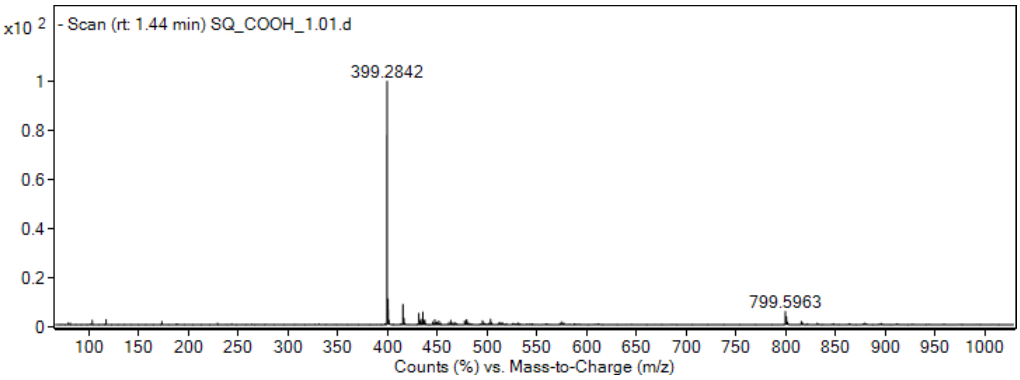

Figure S4. ESI-MS spectrum of SQ-COOH in negative ion mode.

2. Structural characterization of H_2N-PEG_{1500}-NH-Boc

2.1. ^{1}H-NMR spectrum

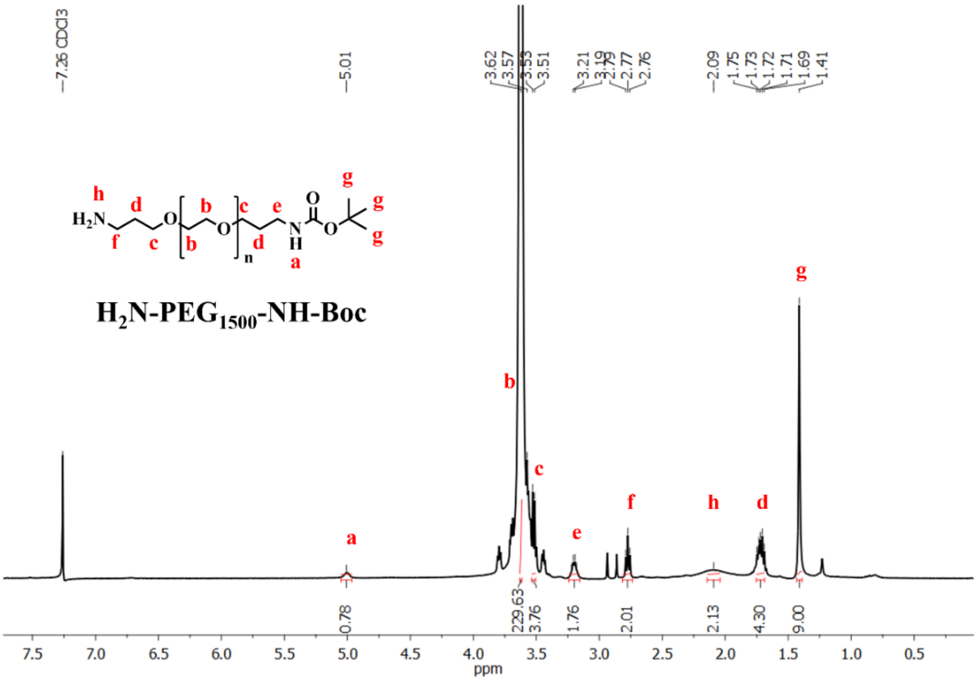

Figure S5. ^{1}H-NMR ($CDCl_3$, 400 MHz) spectrum of H_2N-PEG_{1500}-NH-Boc.

2.2. ^{13}C-NMR spectrum

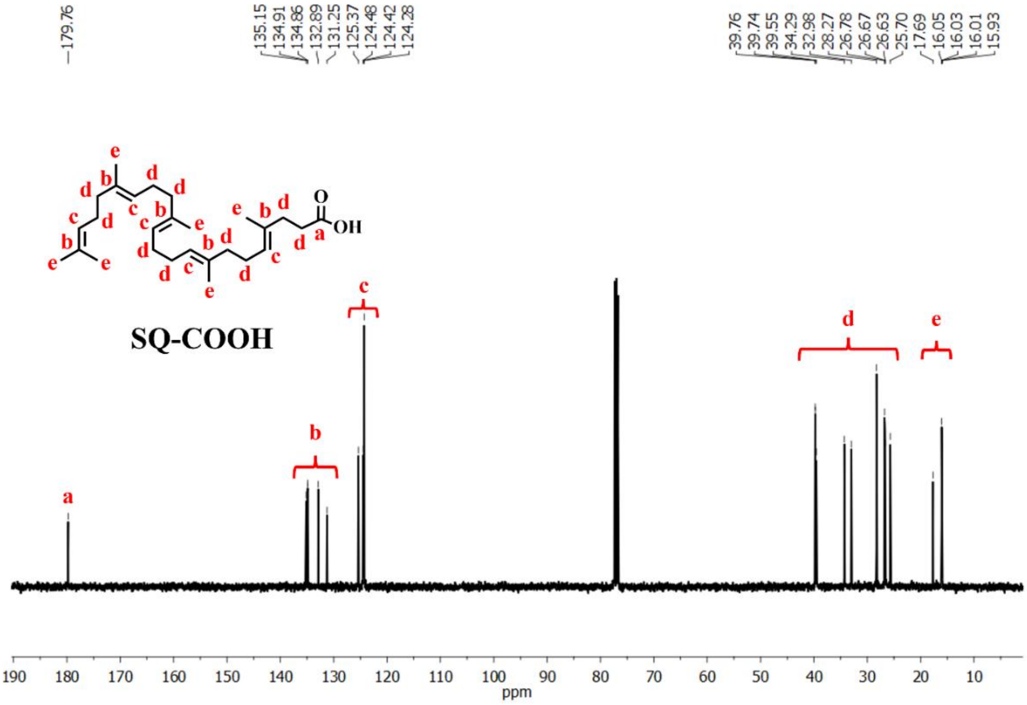

Figure S6. ^{13}C-NMR ($CDCl_3$, 400 MHz) spectrum of H_2N-PEG_{1500}-NH-Boc.

2.3. ATR-FTIR spectrum

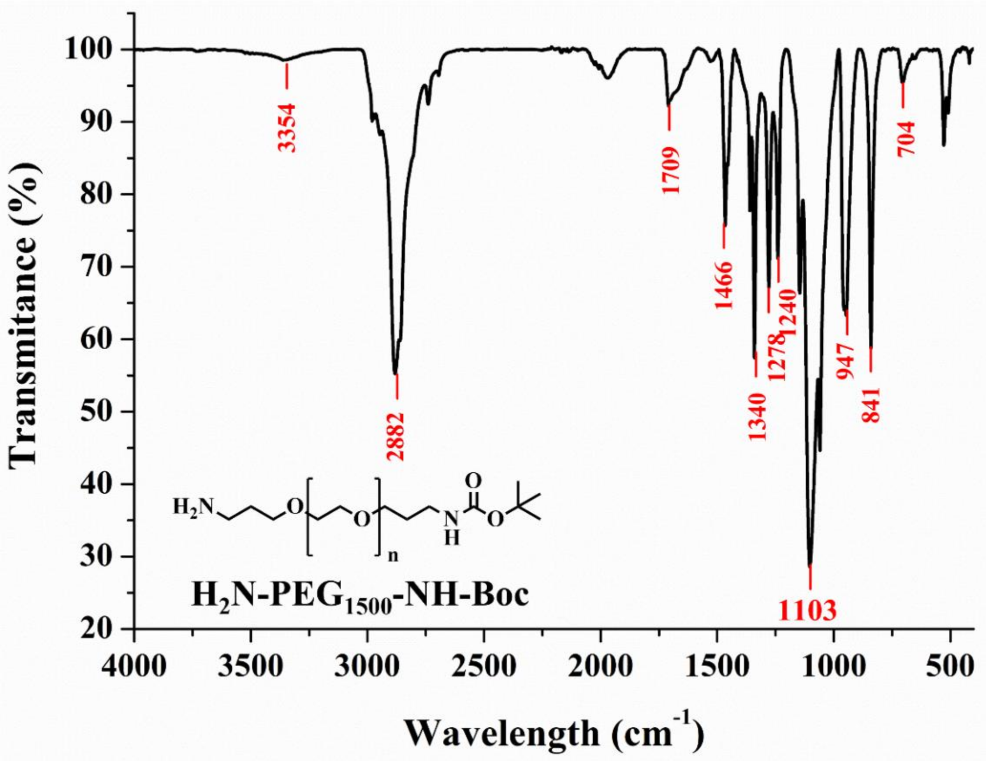

Figure S7. ATR-FTIR spectrum of H_2N-PEG_{1500}-NH-Boc.

2.4. MALDI-TOF/TOF MS spectrum

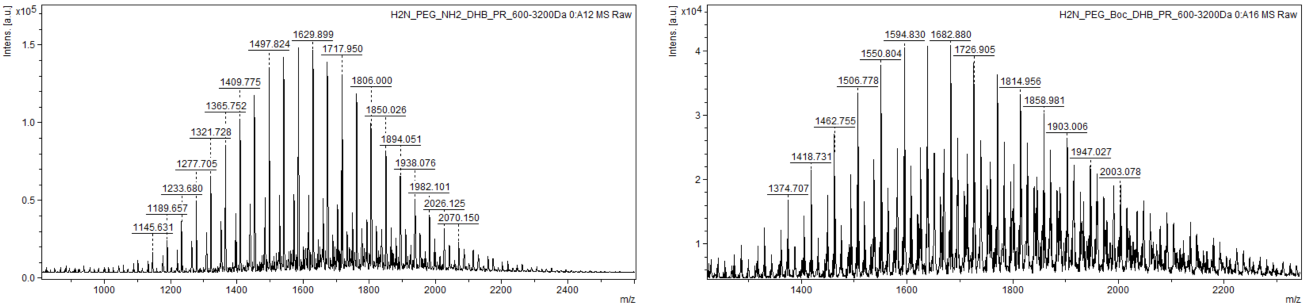

(**a**) (**b**)

Figure S8. MALDI-TOF/TOF MS spectrum of: (**a**) H_2N-PEG_{1500}-NH_2 and (**b**) H_2N-PEG_{1500}-NH-Boc, in positive ion reflector mode (DHB matrix).

3. Structural characterization of SQ-PEG1500-NH-Boc

3.1. ^{1}H-NMR spectrum

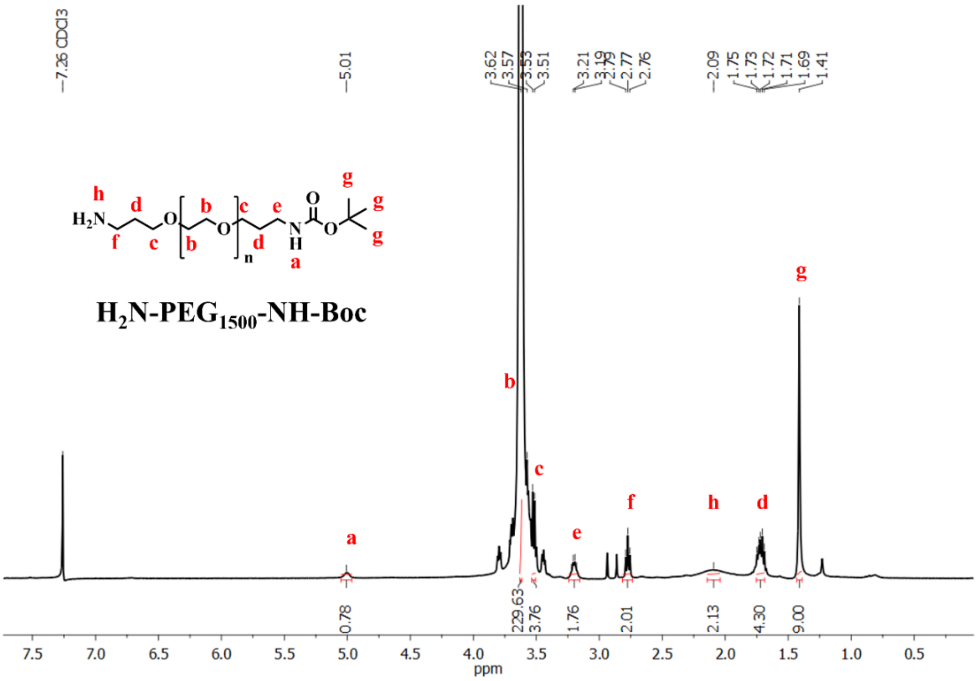

Figure S9. ^{1}H-NMR ($CDCl_3$, 400 MHz) spectrum of SQ-PEG_{1500}-NH-Boc.

3.2. ^{13}C-NMR spectrum

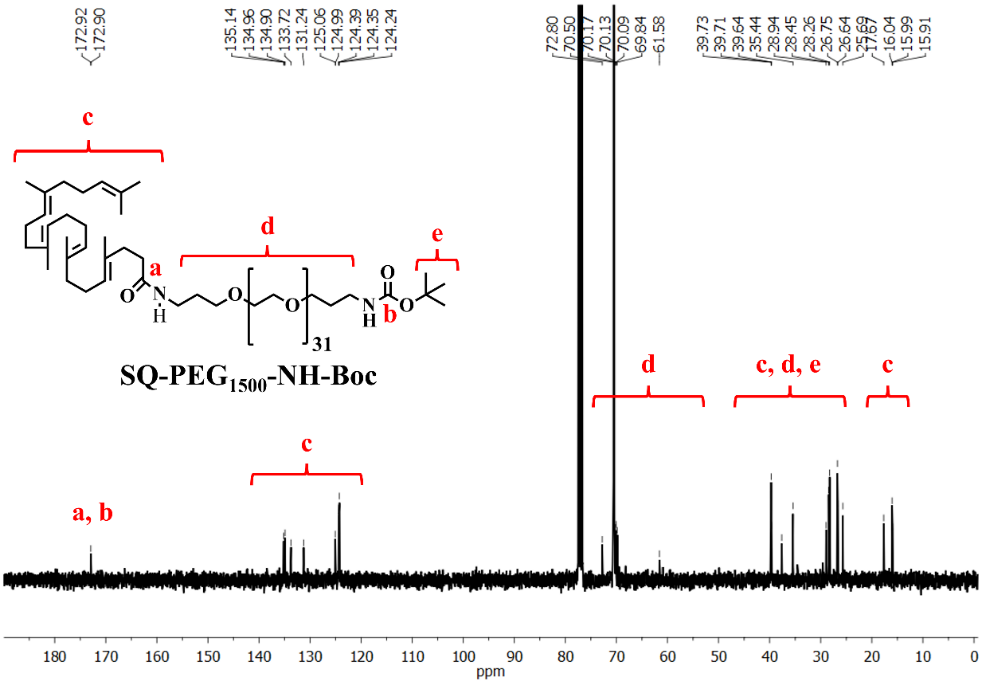

Figure S10. ^{13}C-NMR ($CDCl_3$, 400 MHz) spectrum of SQ-PEG_{1500}-NH-Boc.

3.3. ATR-FTIR spectrum

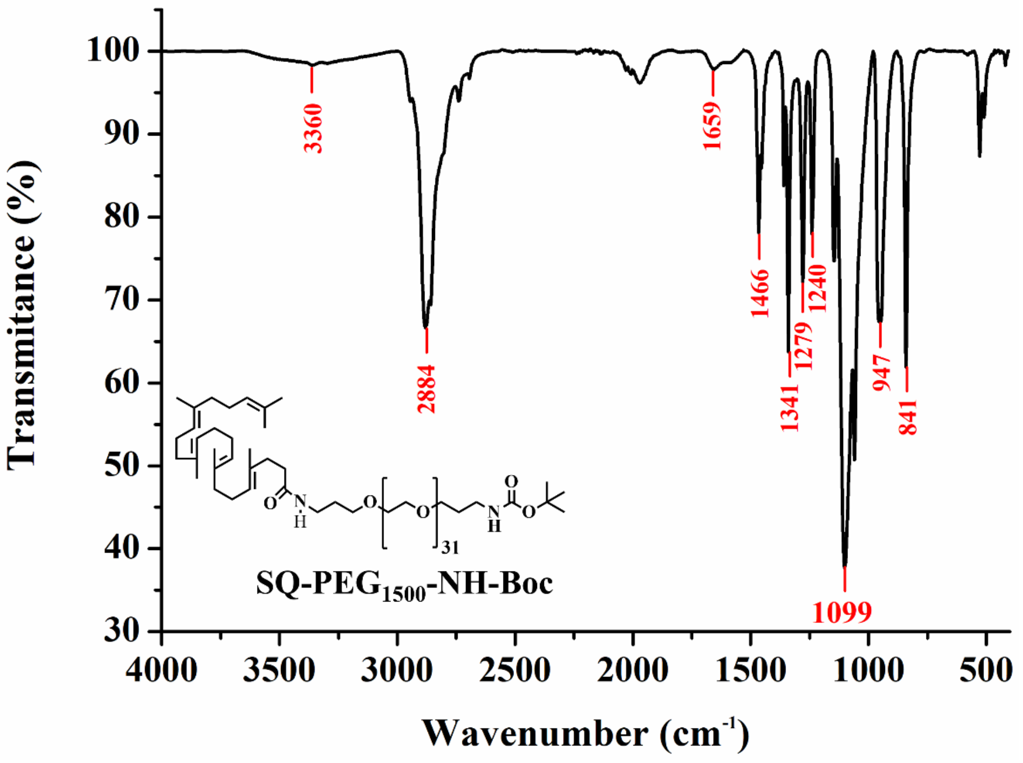

Figure S11. ATR-FTIR spectrum of SQ-PEG$_{1500}$-NH-Boc.

3.4. MALDI-TOF/TOF MS spectrum

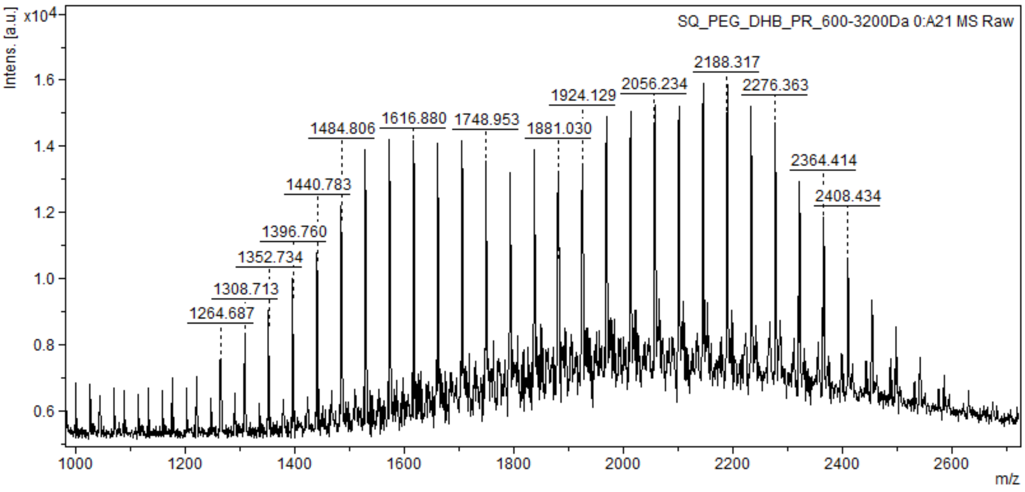

Figure S12. MALDI-TOF/TOF MS spectrum of SQ-PEG$_{1500}$-NH-Boc in positive ion reflector mode (DHB matrix).

4. Methods

4.1. DLS analysis of SQ-PEG$_{1500}$-NH-Boc in PBS solutions

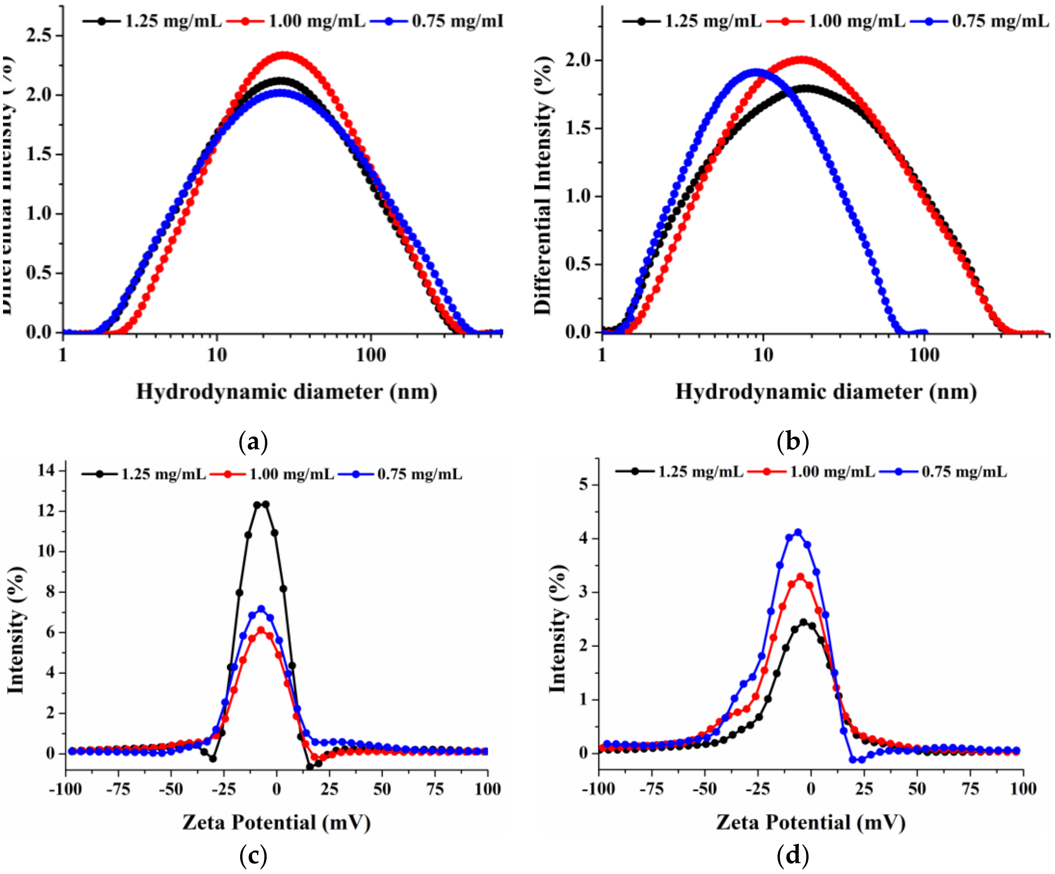

Figure S13. DLS analysis of SQ-PEG$_{1500}$-NH-Boc micelles in PBS solutions at three concentrations (1.25 mg/mL, 1.00 mg/mL, and 0.75 mg/mL). (**a**) Hydrodynamic diameter distributions by intensity in PBS with pH of 6.5; (**b**) hydrodynamic diameter distributions by intensity in PBS with pH of 7.4; (**c**) average Zeta potentials in PBS with pH of 6.5; (**d**) average Zeta potentials in PBS with pH of 7.4.

Table S1. Colloidal characteristics of SQ-PEG$_{1500}$-NH-Boc micelles in PBS solutions at 23°C in three concentrations (C) above CMC value of 0.154 mg/mL (1.25 mg/mL, 1.00 mg/mL, and 0.75 mg/mL).

C (mg/mL)	pH 6.5			pH 7.4		
	[1] Hydrodynamic diameter (nm)	[1] PDI	[1] Zeta Potential (mV)	[1] Hydrodynamic diameter (nm)	[1] PDI	[1] Zeta Potential (mV)
1.25	44.6 ± 4.4	0.440 ± 0.026	−6.54 ± 0.17	36.4 ± 2.6	0.252 ± 0.174	−2.92 ± 0.67
1.00	48.2 ± 4.5	0.402 ± 0.043	−7.16 ± 0.84	36.1 ± 4.2	0.364 ± 0.163	−5.55 ± 0.80
0.75	51.0 ± 0.8	0.497 ± 0.070	−7.52 ± 1.03	13.3 ± 0.1	0.341 ± 0.030	−7.45 ± 0.81

[1] Data are presented as mean ± SD (n = 3)

4.2. Calibration curves of MTx and Cyt in PBS solutions

4.2.1. Calibration curves of MTx

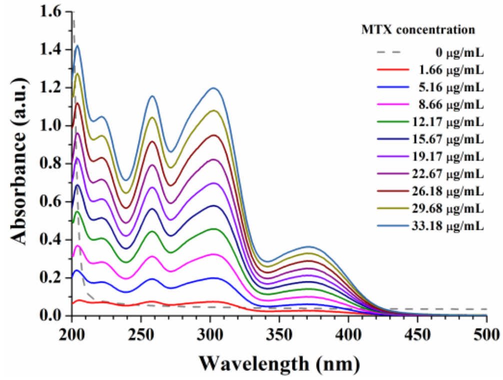

(**a**)

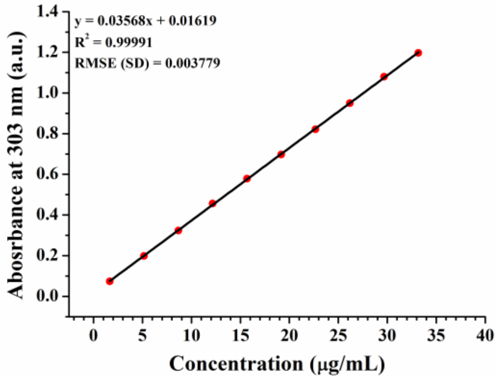

(**b**)

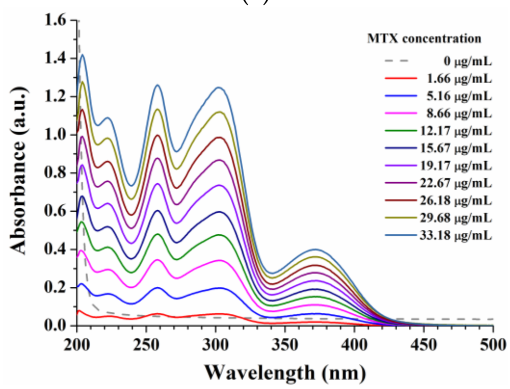

(**c**)

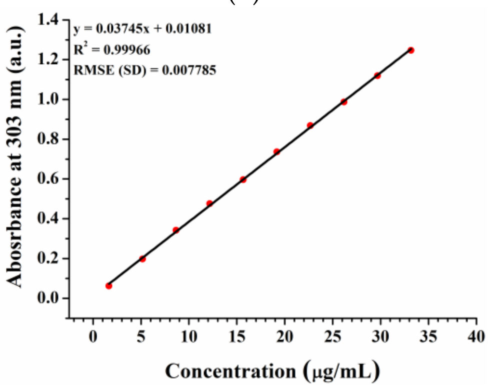

(**d**)

Figure S14. Calibration curves of MTx in PBS by UV-Vis. (**a**) UV-Vis spectra of MTx in PBS with pH 6.5 at concentrations between 0 and 33.18 μg/mL; (**b**) linear fitting of the absorbance recorded at 303 nm as function of MTx concentration (μg/mL) in PBS 6.5; (**c**) UV-Vis spectra of MTx in PBS with pH 7.4 at concentrations between 0 and 33.18 μg/mL; (**d**) linear fitting of the absorbance recorded at 303 nm as function of MTx concentration (μg/mL) in PBS 7.4.

4.2.2. Calibration curves of Cyt

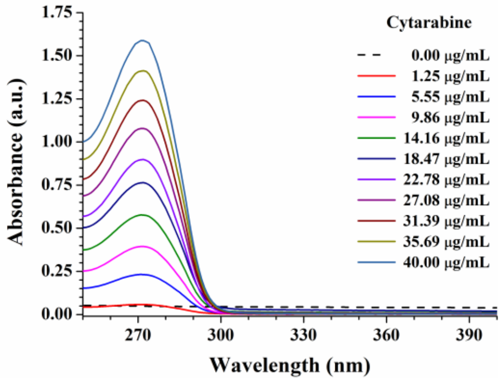

(**a**)

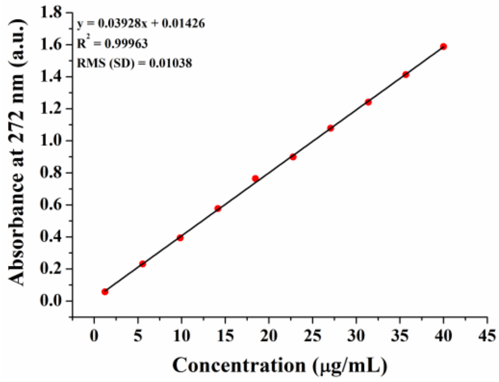

(**b**)

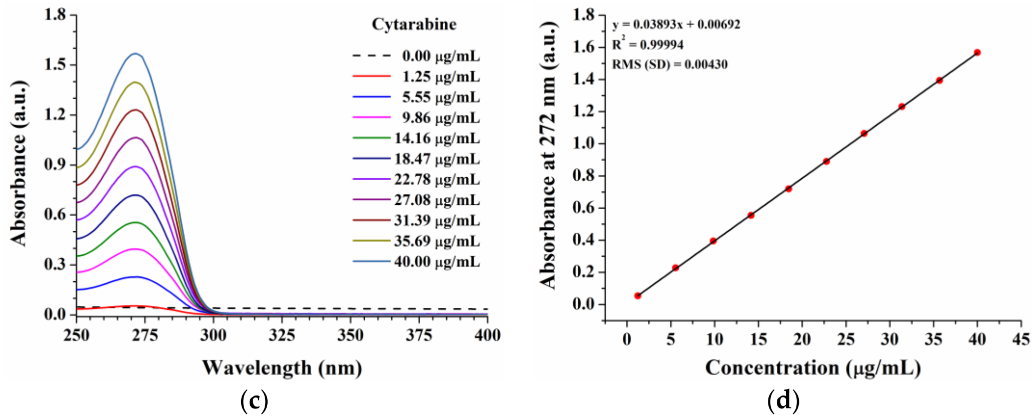

Figure S15. Calibration curves of Cyt in PBS by UV-Vis spectroscopy. (**a**) UV-Vis spectra of Cyt in PBS with pH 6.5 at concentrations between 0 and 40 μg/mL; (**b**) linear fitting of the absorbance recorded at 272 nm as function of Cyt concentration (μg/mL) in PBS 6.5; (**c**) UV-Vis spectra of Cyt in PBS with pH 7.4 at concentrations between 0 and 40 μg/mL; (**d**) linear fitting of the absorbance recorded at 272 nm as function of Cyt concentration (μg/mL) in PBS 7.4.

4.3. DLS analysis of drug-loaded SQ-PEG$_{1500}$-NH-Boc micelles in PBS solutions

4.3.1. DLS analysis of MTx-loaded micelles

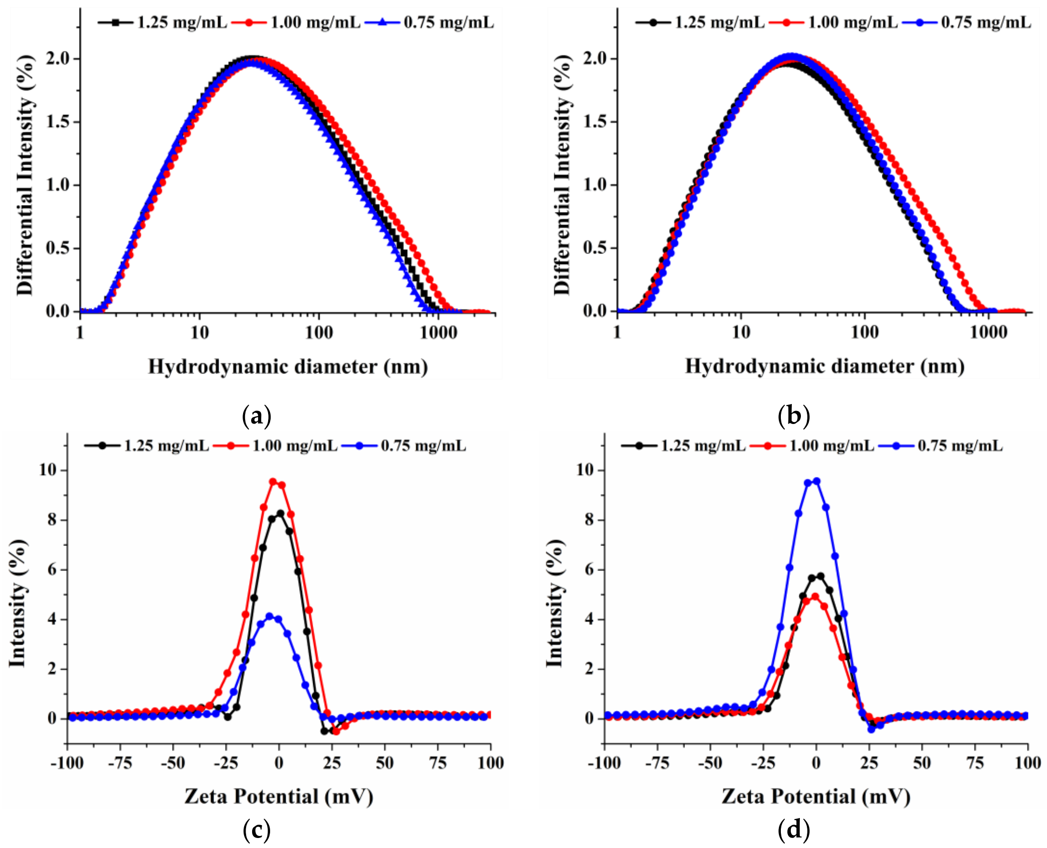

Figure S16. DLS analysis of MTx-loaded SQ-PEG$_{1500}$-NH-Boc micelles in PBS solutions at three concentrations (1.25 mg/mL, 1.00 mg/mL, and 0.75 mg/mL). (**a**) Hydrodynamic diameter distributions by intensity in PBS with pH 6.5; (**b**) hydrodynamic diameter distributions by intensity in PBS with pH 7.4; (**c**) average Zeta potentials in PBS with pH 6.5; (**d**) average Zeta potentials in PBS with pH 7.4.

Table S2. Colloidal characteristics of MTx-loaded SQ-PEG$_{1500}$-NH-Boc micelles in PBS solutions at 23°C in three concentrations (C) (1.25 mg/mL, 1.00 mg/mL, and 0.75 mg/mL).

[2] C (mg/mL)	pH 6.5			pH 7.4		
	[1] Hydrodynamic diameter (nm)	[1] PDI	[1] Zeta Potential (mV)	[1] Hydrodynamic diameter (nm)	[1] PDI	[1] Zeta Potential (mV)
1.25	79.3 ± 5.3	0.303 ± 0.031	−0.43 ± 0.10	56.6 ± 5.9	0.462 ± 0.009	+0.47 ± 0.22
1.00	96.5 ± 8.2	0.299 ± 0.026	−1.14 ± 0.71	76.5 ± 4.4	0.322 ± 0.018	−1.42 ± 0.28
0.75	71.0 ± 3.3	0.573 ± 0.078	−3.69 ± 0.18	59.3 ± 3.5	0.557 ± 0.095	−1.64 ± 0.58

[1] Data are presented as mean ± SD (n = 3).
[2] The concentrations are related to the weight of SQ-PEG1500-NH-Boc without encapsulated MTx.

4.3.2. DLS analysis of Cyt-loaded micelles

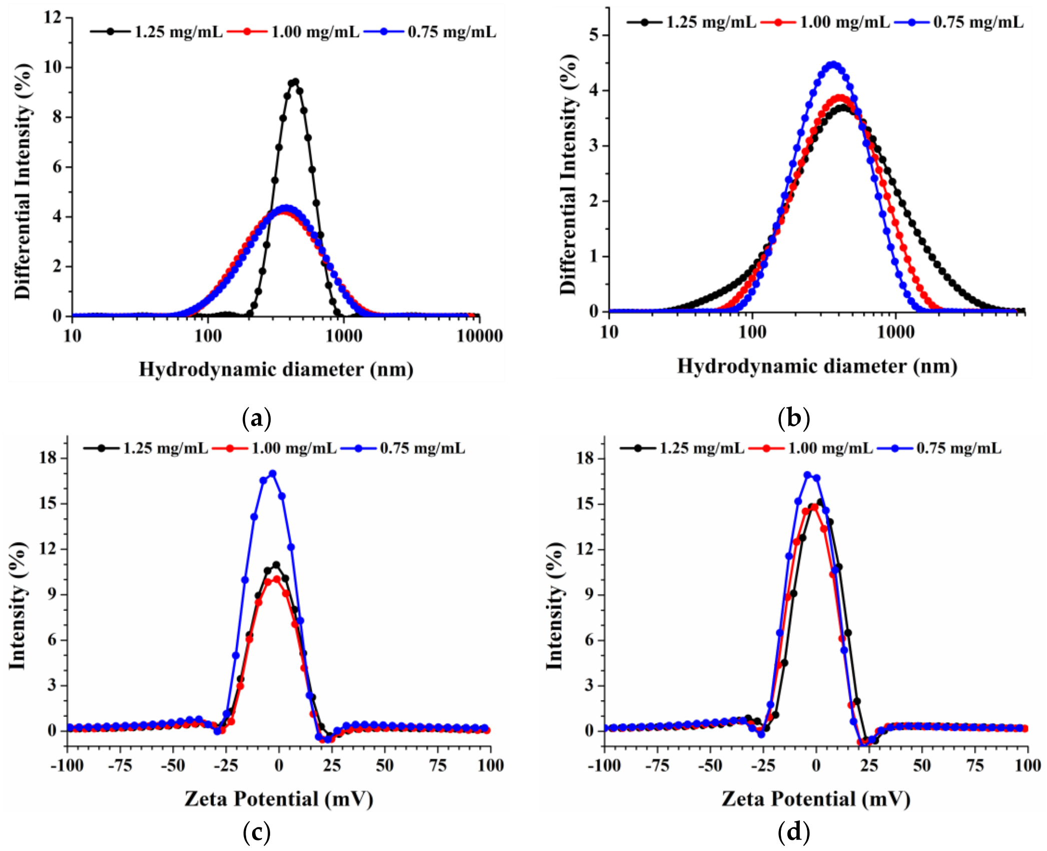

Figure S17. DLS analysis of Cyt-loaded SQ-PEG$_{1500}$-NH-Boc micelles in PBS solutions at three concentrations (1.25 mg/mL, 1.00 mg/mL, and 0.75 mg/mL). (**a**) Hydrodynamic diameter distributions by intensity in PBS with pH 6.5; (**b**) hydrodynamic diameter distributions by intensity in PBS with pH 7.4; (**c**) average Zeta potentials in PBS with pH 6.5; (**d**) average Zeta potentials in PBS with pH 7.4.

Table S3. Colloidal characteristics of Cyt-loaded SQ-PEG$_{1500}$-NH-Boc micelles in PBS solutions at 23°C in three concentrations (C) (1.25 mg/mL, 1.00 mg/mL, and 0.75 mg/mL).

[2] C (mg/mL)	pH 6.5			pH 7.4		
	[1] Hydrodynamic diameter (nm)	[1] PDI	[1] Zeta Potential (mV)	[1] Hydrodynamic diameter (nm)	[1] PDI	[1] Zeta Potential (mV)
1.25	438.9 ± 7.5	0.247 ± 0.021	−2.21 ± 0.11	551.3 ± 5.5	0.260 ± 0.046	+0.70 ± 0.35
1.00	401.1 ± 7.9	0.219 ± 0.011	−2.63 ± 0.93	460.1 ± 5.6	0.186 ± 0.002	−2.10 ± 0.08
0.75	400.6 ± 9.2	0.206 ± 0.012	−4.10 ± 0.29	400.8 ± 1.9	0.186 ± 0.001	−2.56 ± 0.31

[1] Data are presented as mean ± SD (n = 3).
[2] The concentrations are related to the weight of SQ-PEG1500-NH-Boc without encapsulated Cyt.

4.4.The study of MTx and Cyt release kinetics on mathematical models

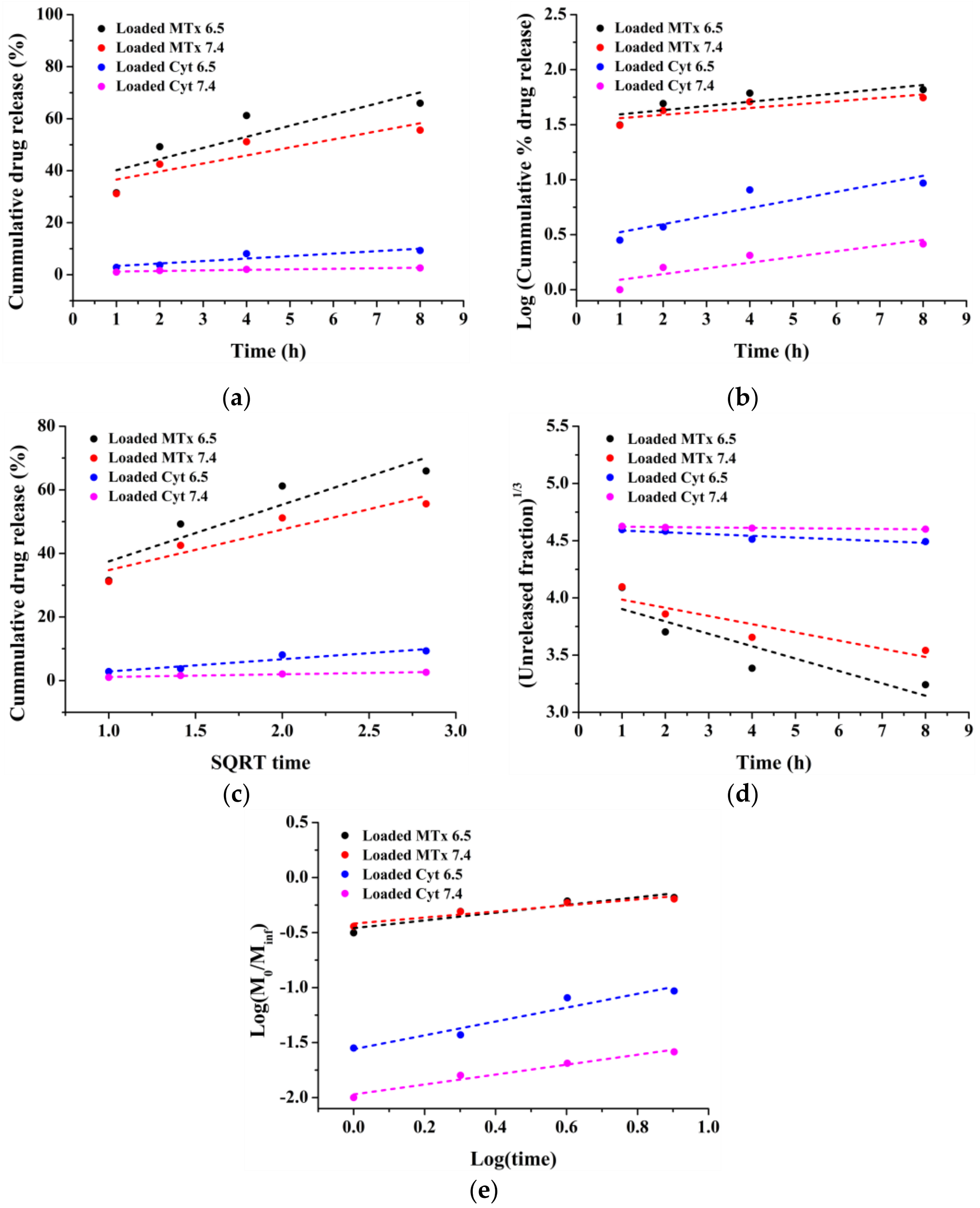

Figure S18. Linear fitting of the mathematical models applied for the release of MTx and Cyt from SQ-PEG1500-NH-Boc micelles: (**a**) Zero-order model; (**b**) First-order model; (**c**) Higuchi model, (**d**) Hixson-Crowell model; and (**e**) Korsmeyer-Peppas model.

4.5. Samples concentration for the in-vitro antitumor experiments

4.5.1. MTx-loaded SQ-PEG1500-NH-Boc micelles

Table S4. Concentrations of the MTx-loaded SQ-PEG_{1500}-NH-Boc micelles which were used for the *in-vitro* antitumor activity experiments calculated as MTx and SQ-PEG_{1500}-NH-Boc. The real concentration for the unloaded micelles is shown in this table, but for the simplicity of the graphic, the concentration shown in figure 11 from the paper is this concentration divided by 38.92 which is representing the weight ratio between MTx and SQ-PEG_{1500}-NH-Boc.

Compound	Concentration (µg/mL)							
MTx	0.78	1.56	3.13	6.25	12.5	25	50	100
SQ-PEG_{1500}-NH-Boc	30.36	60.73	121.85	243.30	486.61	973.21	1946.43	3892.86

4.5.2. Cyt-loaded SQ-PEG_{1500}-NH-Boc micelles

Table S5. Concentrations of the Cyt-loaded SQ-PEG_{1500}-NH-Boc micelles which were used for the *in-vitro* antitumor activity experiments calculated as Cyt and SQ-PEG_{1500}-NH-Boc. The real concentration for the unloaded micelles is shown in this table, but for the simplicity of the graphic, the concentration shown in figure 12 from the paper is this concentration divided by 16.97 which is representing the weight ratio between Cyt and SQ-PEG_{1500}-NH-Boc.

Compound	Concentration (µg/mL)					
Cyt	0.63	1.25	2.50	5.00	10.00	20.00
SQ-PEG_{1500}-NH-Boc	10.69	21.20	42.41	84.82	169.63	339.27
